# Development of Functional Synbiotic-Designed Sorbets as Carriers of *Lactiplantibacillus plantarum* 299v and Fruit-Derived Bioactive Compounds: Probiotic Survival, Physicochemical Quality, Consumer Acceptance, and Purchase Intention

**DOI:** 10.3390/molecules31183258

**Published:** 2026-09-14

**Authors:** Agnieszka Palka, Marianna Palka

**Affiliations:** 1 Department of Quality Management, Faculty of Management and Quality Science, Gdynia Maritime University, 81-225 Gdynia, Poland; 2 Faculty of Mathematics, Physics and Informatics, University of Gdańsk, 80-309 Gdańsk, Poland; marianna.palka02@gmail.com

**Keywords:** functional food, functional synbiotic-designed sorbet, *Lactiplantibacillus plantarum* 299v, inulin, probiotics, prebiotics, fruit-derived bioactive compounds, texture

## Abstract

The growing demand for dairy-free functional foods has created a need for fruit-based products that simultaneously provide probiotics, prebiotics, and bioactive compounds. This study evaluated seven high-fruit synbiotic-designed sorbets prepared with raspberries, strawberries, haskap berries, apples, or plums, and two variants with blackcurrants, containing 45–55% fruit, 3% inulin, and *Lactiplantibacillus plantarum* 299v added at two inoculum levels. Sucrose was added to the mixture, and in one variant of the blackcurrant, it was replaced with erythritol, which can affect, among other things, the sweetness, hardness, melting point, mouthfeel, and freezing point of frozen desserts. Erythritol was used to evaluate the effect on these characteristics in one variant. The addition of one and two probiotic capsules to each sorbet was also compared. The viability of the probiotics (as LAB) before and after freezing, physicochemical composition, color, hardness, melting, aeration, antioxidant properties, consumer acceptance, and purchase intention were assessed using univariate and multivariate statistical analyses. After freezing, the number of viable cells remained at approximately 7.05–7.18 log CFU/g in the single-capsule variants and 7.45–7.51 log CFU/g in the two-capsule variants, confirming the high immediate viability of the probiotic cells. The fruit type and its content in the matrix significantly differentiated acidity, sugars, color, hardness, degree of growth, melting properties, phenolic compounds, anthocyanins, antioxidant activity, and sensory quality. The dark berry sorbets showed a particularly high content of bioactive compounds. Consumer responses varied significantly between formulations, with taste, texture, and consistency demonstrating the strongest associations with overall acceptance. The results demonstrate that fruit sorbets can serve as practical, dairy-free carriers for *Lactiplantibacillus plantarum* 299v and inulin, while retaining fruit-specific functional and sensory properties.

## 1. Introduction

Functional food development increasingly focuses on matrices that combine nutritional value, sensory appeal, and bioactive ingredients. Frozen fruit desserts are promising because they are dairy-free, widely accepted, rich in phytochemicals, and can incorporate prebiotic carbohydrates without the fat typical of ice cream [1,2]. Fruit-based frozen desserts, without added milk, can be a better source of nutritional value than dairy ice cream. They do not contain milk proteins, which can cause food allergies, and they do not contain lactose, which many consumers are intolerant to. Therefore, they can be an excellent source of health-promoting, probiotic and synbiotic ingredients for a larger group of consumers than dairy ice cream.

A synbiotic is defined as a mixture of live microorganisms and substrate(s) selectively utilized by host microorganisms that confers a health benefit [3]. Complementary synbiotics combine established probiotics and prebiotics, whereas synergistic synbiotics are designed for selective substrate use by the co-administered microorganism. Thus, a probiotic-inulin product may be considered a candidate complementary synbiotic, but a definitive claim requires evidence of host benefit [3]. Development of a synbiotic food requires both technological evidence that viable cells can be delivered and biological justification for the selected microorganism-substrate combination [3,4,5,6,7]. Most commercial probiotic foods are dairy-based, and milk proteins, lactose, fat globules, and buffering capacity can protect microbial cells. However, these products are unsuitable for consumers with lactose intolerance, milk-protein allergy, vegan diets, or other preferences. This has increased interest in plant matrices made from fruits, vegetables, cereals, legumes, and soy. Although these matrices provide carbohydrates, fiber, vitamins, minerals, and phenolics, they may also expose microorganisms to low pH, weak buffering, oxygen, osmotic stress, and the absence of a protective fat-protein network [7,8,9]. Although dairy food matrices remain at the forefront of probiotic delivery, certain non-dairy plant-based food matrices (i.e., fruits) have been used as successful carriers in delivering probiotics to humans. Probiotics, in particular lactobacilli and bifidobacteria in these products, demonstrated their ability to maintain adequate viable probiotic numbers (10^6^–10^8^ CFU/mL or g of the carrier food product) during product shelf life [9]. Frozen desserts can extend shelf life by limiting microbial metabolism, but freezing may damage probiotic cells through ice-crystal formation, solute concentration, dehydration, membrane transitions, mechanical injury, oxidative stress, and temperature fluctuations. Survival depends on the strain, inoculum preparation, freezing and thawing conditions, storage temperature, total solids, sugars, proteins, lipids, hydrocolloids, and prebiotic fibers; therefore, results cannot be transferred reliably between matrices [10,11,12]. Probiotic and synbiotic sorbets remain less studied than dairy ice cream or frozen yogurt. Existing studies usually concern one matrix, one or two strains, and a limited set of microbiological, physicochemical, or sensory outcomes. Pumpkin desserts with *Lacticaseibacillus rhamnosus* and inulin, fruit-tea sorbets with oligofructose, and *jussara* sorbets containing polydextrose, *Lactobacillus acidophilus* LA3, and *Lacticaseibacillus paracasei* BGP1 demonstrated that plant-based frozen matrices can maintain probiotic populations and acceptable quality [9,13,14]. Other paper on mango, cocoa, microencapsulation, and fermented frozen desserts shows trade-offs. Encapsulation may improve survival while causing graininess, and fermentation may increase acidity and reduce acceptance despite better viability or antioxidant properties. Accordingly, survival, structure, sensory quality, and consumer response should be evaluated together [12,15]. Research specifically involving *Lactiplantibacillus plantarum* 299v in non-dairy frozen products is limited. An avocado-banana dessert maintained probiotic counts and sensory quality for eight weeks, but its protective lipid- and polysaccharide-rich matrix differs from a high-water, fat-free sorbet [7]. *Lactiplantibacillus plantarum* 299v is well characterized, with documented acid and bile tolerance, and interactions with barrier and immune functions [16,17,18,19]. Clinical studies reported a positive influence on health and the digestive tract, especially the gut [19,20,21,22,23,24]. Evidence from human intervention studies indicates the safety of consuming *L. plantarum* 299v in amounts up to 2 × 10^11^ CFU/day [25]. Maintaining viable *Lactiplantibacillus plantarum* 299v cells through manufacture and freezing is therefore essential. Inulin is a β-(2→1)-linked fructan that resists human digestion and reaches the colon for microbial fermentation and the metabolites may support metabolism, barrier function, and immune signaling [3,4,26,27]. Inulin can improve growth, activity, or survival of probiotics [5,6,8,21,22,28]. Technologically, it binds water, increases solids and viscosity, and can influence overrun, hardness, melting, and ice recrystallization. Benefits have been reported in frozen and synbiotic ice cream [11,29], while 2% and 5% additions changed viscosity, melting, antioxidant-related measures, and sensory quality in fruit sorbets [2]. The fixed 3% of inulin used in this study may provide prebiotic fiber and stabilize a fat-free matrix.

Fruits differ in vitamins, acids, pectins, sugars, minerals, and polyphenols. Strawberry provides, i.e., vitamin C, anthocyanins, flavonols, phenolic acids [30,31,32]. Raspberry supplies anthocyanins, ellagitannins, flavonols, fiber, and ellagic-acid precursors [33,34]. Haskap berry contains anthocyanins, phenolic acids, flavonols, proanthocyanidins, vitamin C, and iridoids [35,36,37]. Blackcurrant is rich in vitamin C, anthocyanins, flavonols, phenolic acids, and proanthocyanidins [38,39]. Apple provides pectin, quercetin derivatives, flavanols, procyanidins, chlorogenic acid, and phloridzin, many concentrated in the peel [40,41,42]. Plums contain fiber, organic acids, hydroxycinnamic acids, flavonols, and anthocyanins [43,44]. Functional value is relevant only when consumers accept the product. Probiotic or prebiotic enrichment may alter acidity, sweetness, aroma, mouthfeel, melting, hardness, and aftertaste. In synbiotic ice cream, fermentation by *Lactiplantibacillus plantarum* or *Lactobacillus casei* can improve selected technological and antioxidant properties but reduces overall acceptance when acidity becomes perceptible. Inulin may improve creaminess and melting, although excessive amounts can create atypical body or sweetness [12,14,29]. Purchase intention extends hedonic assessment by integrating perceived quality, familiarity, expected health value, price sensitivity, dietary preferences, and willingness to adopt a novel food, yet it has rarely been analyzed together with probiotic survival, instrumental properties, inulin, and fruit bioactives in sorbets.

Current evidence indicates that probiotics can survive in selected frozen desserts, inulin can support microbial and structural stability, and fruit sorbets can retain antioxidant compounds. However, these research areas have largely developed separately. Studies of probiotic or synbiotic sorbets are few, typically use a single matrix or different strains, and often omit comprehensive physicochemical, sensory, and purchase-intention analyses. Research on *Lactiplantibacillus plantarum* 299v has focused mainly on supplements, beverages, minimally processed foods, or an avocado-based frozen dessert. Consequently, little is known about the behavior of one clinically characterized strain across several high-fruit, fat-free matrices produced under a common formulation framework.

When designing a functional, synbiotic food product, it’s important to consider not only its physicochemical properties but also its sensory properties, along with consumer sensory evaluation and potential purchase intentions. It’s the consumer who decides whether a product will find a buyer. The combined use of physicochemical and microbiological testing, along with consumer research, is a new approach to researching functional, synbiotic-designed sorbets. This study was the first to integrate four dimensions of the research: potential probiotic value, assessed by viable LAB/*Lactiplantibacillus*, most probably *Lactiplantibacillus plantarum* 299v counts (before and after freezing and by comparison of two inoculum levels; prebiotic and technological value, provided by a constant 3% inulin addition; fruit-derived health-promoting value, characterized using antioxidant-related compounds and physicochemical indices; and consumer relevance, evaluated through sensory acceptance and purchase intention. Seven formulations based on strawberry, raspberry, haskap berry, two blackcurrant variants, apple, and plum enabled comparison of matrix effects while maintaining a common high-fruit product concept. The aim was to develop and comprehensively evaluate these synbiotic-designed, functional sorbets and to determine whether replacing sucrose with erythritol in one blackcurrant formulation modified composition, freezing behavior, and consumer response while maintaining probiotic viability and bioactive quality. The additional goal was to develop a production method that could be easily reproduced, even at home. The hypotheses were: H1, *Lactiplantibacillus plantarum* 299v remains technologically viable after freezing in all matrices at level at least 6 log CFU/g; H2, addition of two capsules of *Lactiplantibacillus plantarum* 299v results higher post-freezing counts than one capsule, with matrix-dependent survival; H3, consumer acceptance and purchase intention differ among formulations and depend mainly on taste, consistency, structure, appearance, and color; and H4, erythritol alters melting, hardness, sweetness, consumer evaluation and purchase intention while preserving fruit bioactives and viable 299v. Using one strain and one inulin level across contrasting fruits was intended to distinguish general process effects from matrix-specific responses and support evidence-based selection of non-dairy probiotic matrices.

## 2. Results

**Figure 1:**
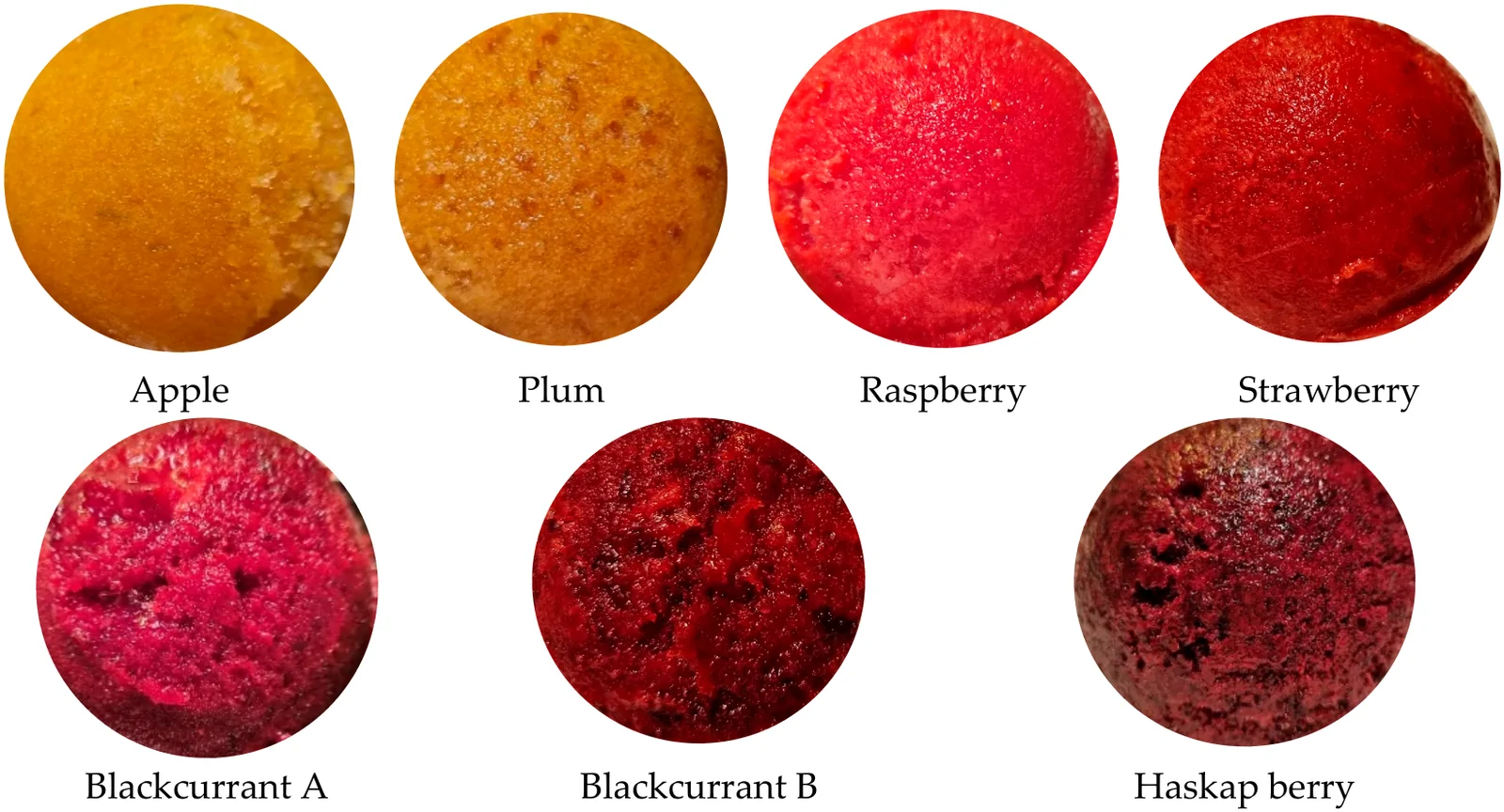
Sorbets prepared for this study.

The results of this study were described in several subsections, starting with physicochemical parameters, then *Lactiplantibacillus plantarum* 299v amount (evaluated as LAB/*Lactiplantibacillus*), freezing survival analysis, instrumental color analysis, and consumer acceptance and purchase willingness analysis at the end of this chapter. Statistical analysis of all results was conducted to identify associations, correlations, and other relationships between all assessed factors. Pictures of all sorbets are shown in Figure 1.

### 2.1. Physicochemical and Color Parameters of the Sorbets

Physicochemical parameters of sorbets (mean ± SD; Tukey groups) are given in Table 1a–d. The investigated sorbets differed significantly in most of the analyzed physicochemical, chemical, textural, and color-related properties. The results indicate that the type of fruit (as well as the amount in the matrix) used and, in the case of blackcurrant B sorbet, the replacement of sucrose with erythritol, markedly influenced acidity, antioxidant potential, sugar composition, total solids content, texture, melting behavior, and product color.

The pH values of the analyzed sorbets (Table 1a) ranged from 2.74 to 3.58, confirming the strongly acidic character of all products. The lowest pH was assessed for blackcurrant B sorbet, at 2.74, whereas the highest value was observed for strawberry sorbet, at 3.58. This difference was statistically significant. Raspberry, blackcurrant A, and plum sorbets showed similar pH values of 2.89, 2.86, and 2.90, respectively, and belonged to the same homogeneous group. Haskap berry and apple sorbets showed intermediate pH values of 3.20 and 3.18, respectively. A particularly interesting observation was obtained when comparing the two blackcurrant sorbet formulations. Blackcurrant B had a significantly lower pH than blackcurrant A, although the titratable acidity values of both products were almost identical. This may indicate that the use of erythritol instead of sucrose altered the ionic equilibrium or buffering properties of the matrix without significantly affecting the total amount of acids determined by titration.

Potential acidity (Table 1a) ranged from 2.76 to 9.43°SH. The highest value was recorded for haskap berry sorbet, at 9.43°SH, and was significantly higher than that of all other formulations. The lowest potential acidity was assessed in strawberry sorbet, at 2.76°SH, which was consistent with its highest pH value. Raspberry, blackcurrant A, blackcurrant B, and plum sorbets formed a common statistical group, with values ranging from 5.46 to 5.57°SH.

These results confirm that pH and titratable acidity describe different aspects of product acidity. Haskap berry sorbet provides a clear example: it did not have the lowest pH, but it exhibited the highest potential acidity. This indicates the presence of a large pool of dissociated and undissociated organic acids as well as compounds with buffering capacity. Such characteristics may affect not only the perception of sourness but also microbiological stability and the stability of bioactive compounds.

The antiradical activity of the sorbets varied considerably (Table 1a), ranging from 57.56 to 93.35%. The highest value was obtained for haskap berry sorbet, at 93.35%. Very high activity was also recorded for both blackcurrant sorbets: 92.47% for blackcurrant A and 91.66% for blackcurrant B. The values obtained for these three formulations did not differ significantly. Raspberry sorbet showed an antiradical activity of 90.24% and partially overlapped in terms of homogeneous group classification with both the highest-activity formulations and strawberry sorbet. The antiradical activity of strawberry sorbet was 86.45%. Considerably lower values were recorded for plum and apple sorbets, at 65.54% and 57.56%, respectively. Apple sorbet showed the lowest free-radical scavenging capacity and differed significantly from all berry-based formulations. The results of antioxidant activity determined by the FRAP assay indicated significant differences among the analyzed products. The highest values were evaluated for blackcurrant B and A, and haskap berry, at 33.09, 28.39, 26.17 µmol TE/g respectively. An increase in antioxidant activity was observed with increasing polyphenol and anthocyanin content.

However, the highest total polyphenol content was observed not in haskap berry sorbet but in plum sorbet, at 7.59 mg GAE/g (Table 1a). Raspberry sorbet had the second-highest polyphenol content, at 7.04 mg GAE/g, followed by haskap berry sorbet, at 6.55 mg GAE/g. The lowest total polyphenol content was recorded in apple sorbet, at 3.95 mg GAE/g. The two blackcurrant formulations contained almost identical amounts of polyphenols, at 5.45 and 5.44 mg GAE/g, and belonged to the same statistical group.

The lack of complete agreement between total polyphenol content and antiradical activity indicates that the antioxidant potential of the sorbets was not determined solely by the overall quantity of phenolic compounds. Plum sorbet was the most evident example, as it had the highest total polyphenol content but only moderate antiradical activity. In contrast, blackcurrant sorbets contained fewer total polyphenols than plum sorbet but exhibited considerably higher antiradical activity. This may be attributed to qualitative differences in the phenolic profiles. Individual polyphenols differ in the number and position of hydroxyl groups, degree of polymerization, bioavailability, and ability to donate hydrogen atoms or electrons. The measured antioxidant activity may also have been influenced by vitamin C, anthocyanins, and other reducing compounds present in the fruit matrix. Therefore, total polyphenol content and antiradical activity should be considered complementary rather than interchangeable parameters [45,46,47].

The highest anthocyanin content was recorded in haskap berry sorbet, at 1808.3 mg/100 g (Table 1a). This value was significantly higher than those obtained for all other formulations. Both blackcurrant sorbets contained 1294.3 mg/100 g anthocyanins and formed a common homogeneous group. The absence of a difference between blackcurrant A and blackcurrant B indicates that replacing sucrose with erythritol did not affect the measured anthocyanin content. Considerably lower anthocyanin levels were found in raspberry and strawberry sorbets, at 166.0 and 144.0 mg/100 g, respectively. The lowest value was recorded in plum sorbet, at 86.0 mg/100 g, whereas apple sorbet contained 125.3 mg/100 g. The high anthocyanin content of haskap berry and blackcurrant sorbets corresponded with their very dark color, low L* values, and high antiradical activity. Exploratory analysis showed a clear decrease in product lightness with increasing anthocyanin content. This indicates that anthocyanin accumulation was one of the main factors responsible for the dark and intense color of these sorbets. At the same time, plum sorbet, despite having the highest total polyphenol content, contained the lowest amount of anthocyanins and showed markedly lower antiradical activity than haskap berry and blackcurrant sorbets. This may suggest that, in the analyzed products, not only the total quantity of polyphenols but also their composition, including the proportion of anthocyanins, was particularly important for free-radical scavenging capacity.

Vitamin C content differed significantly among the formulations and ranged from 3.52 to 94.78 mg/100 g (Table 1a). The highest value was found in haskap berry sorbet, at 94.78 mg/100 g. This was followed by blackcurrant A and blackcurrant B sorbets, which contained 65.33 and 51.72 mg/100 g, respectively. The difference between the two blackcurrant formulations was statistically significant, despite their identical anthocyanin content and almost identical total polyphenol content. Lower vitamin C levels were recorded in strawberry sorbet, at 42.82 mg/100 g, and raspberry sorbet, at 16.07 mg/100 g. Apple and plum sorbets contained the lowest amounts of vitamin C, at 8.58 and 3.52 mg/100 g, respectively. The pattern of vitamin C content was largely consistent with the results of antiradical activity. Haskap berry and both blackcurrant sorbets were characterized by the highest vitamin C levels and the highest antiradical activity. Apple and plum sorbets contained the least vitamin C and exhibited the lowest or moderate antiradical activity. This suggests that ascorbic acid may have contributed substantially to the total antioxidant potential of the products. The comparison between plum and haskap berry sorbets is particularly noteworthy. Plum sorbet contained more total polyphenols but substantially less vitamin C and fewer anthocyanins, while its antiradical activity was almost 28 percentage points lower. This observation supports the importance of synergistic interactions among different groups of antioxidants.

The hardness values ranged from 5628.3 to 7287.3 N (Table 1b). Blackcurrant B was the hardest sorbet, with a value of 7287.3 N, and differed significantly from all other formulations. The lowest hardness was recorded for apple sorbet, at 5628.3 N. Raspberry and haskap berry sorbets showed intermediate and statistically similar values of 6509.3 and 6560.7 N, respectively. Strawberry, blackcurrant A, and plum sorbets belonged to the same homogeneous group. The comparison of blackcurrant A and blackcurrant B clearly demonstrates that the type of sweetener strongly affected the structure of the frozen product. Replacing sucrose with erythritol increased hardness from 6280.0 to 7287.3 N, corresponding to an increase of approximately 16%. At the same time, blackcurrant B had the lowest total solids content, the lowest soluble solids content, and the lowest overrun. This combination may have resulted in the formation of a denser and less aerated structure. Sucrose contributes to freezing-point depression, the amount of unfrozen aqueous phase, and product softness [48,49,50]. Its absence from blackcurrant B, despite the presence of erythritol, may have altered water crystallization and increased the proportion of the frozen phase. The lower amount of incorporated air may have additionally reduced the mechanical softening effect associated with aeration. The results therefore indicate that sorbet hardness was determined not only by the total amount of dissolved solids but also by their type and by the degree of aeration.

Melting resistance shows the quality of the sorbet mixture used in production and changes during storage time; it shouldn’t be higher than 50%. The values of the melting resistance ranged from 14.67 to 30.47%; therefore, it can be assumed that this parameter indicated high quality of sorbet matrices (Table 1b). The highest value was obtained for apple sorbet, at 30.47%, whereas the lowest was recorded for haskap berry sorbet, at 14.67%. Blackcurrant A had a value of 18.73%, while blackcurrant B reached 25.87%. The results showed clear differences in the melting stability of the analyzed matrices. Haskap berry sorbet, which had the highest total solids content, showed the lowest value of melting resistance, whereas apple sorbet, despite its relatively high total solids content, showed the highest value. This indicates that melting behavior was determined not only by total solids but also by sugar composition, fiber, fruit cell-wall components, water-binding capacity, and the microstructure of the frozen phase.

Overrun is the content of air forced into the sorbet mixture during freezing and depends strictly on the type of freezer and the freezing parameters, as well as the composition of the matrix. Overrun of the sorbets ranged from 19.20 to 36.03% (Table 1b). The highest values were recorded for raspberry and strawberry sorbets, at 36.03 and 35.57%, respectively. A high overrun was also found in apple sorbet, at 34.87%. Blackcurrant B had the lowest overrun, at 19.20%, and differed significantly from all other formulations. Haskap berry and plum sorbets reached 24.90 and 28.70%, respectively. The low overrun of blackcurrant B probably contributed to its high hardness. Air bubbles interrupt the continuity of the ice matrix and reduce the force required to deform the product. In a poorly aerated product, the structure is more compact, and the amount of solid phase per unit volume is greater. The simultaneous occurrence of the lowest overrun and the highest hardness in blackcurrant B is therefore technologically justified. However, this relationship was not completely linear across all formulations. Apple sorbet, which had a high overrun, was the softest product, supporting the above interpretation, whereas raspberry sorbet also had a high overrun but was harder than strawberry sorbet. This suggests that sugar composition, soluble solids, and the properties of insoluble fruit components also contributed to texture.

The highest total solids content was found in haskap berry sorbet, at 34.53% (Table 1b). Apple and plum sorbets contained 32.63 and 32.55% total solids, respectively, and did not differ significantly. The lowest value was recorded for blackcurrant B, at 25.52%, which was 4.68 percentage points lower than that of blackcurrant A. The difference between the blackcurrant formulations confirms the significant effect of replacing sucrose with erythritol on the amount of measured solid substances. The low total solids content of blackcurrant B occurred together with the lowest soluble solids content, the lowest overrun, and the highest hardness. This combination suggests that the formulation produced a less aerated and more compact structure with a high proportion of frozen water. The highest fiber content was recorded in strawberry sorbet, at 1.12 g, followed by plum sorbet, at 0.94 g (Table 1b). The lowest values were found in the two blackcurrant formulations, at 0.54–0.55 g. Raspberry sorbet showed an intermediate value, while haskap berry and apple sorbets did not differ significantly from several adjacent statistical groups. Haskap berry sorbet had the highest ash content, at 0.52% (Table 1b). Strawberry sorbet formed the second-highest group, with a value of 0.37%. Apple sorbet contained the least ash, at 0.14%. Raspberry, both blackcurrant formulations, and plum sorbets contained between 0.22 and 0.25% ash and did not differ significantly. The high total solids, soluble solids, glucose, and ash contents of haskap berry sorbet indicate a high concentration of naturally occurring fruit components. This product was also distinguished by the highest vitamin C and anthocyanin contents, emphasizing its high nutritional and functional value [35,36].

The soluble solids content ranged from 17.87 to 35.80 °Brix (Table 1c). Haskap berry sorbet had the highest value, at 35.80 °Brix, followed by strawberry sorbet, at 30.30 °Brix. Blackcurrant B showed the lowest value, at 17.87 °Brix, which was significantly lower than that of blackcurrant A, at 18.77 °Brix. Raspberry, apple, and plum sorbets showed similar values of approximately 22 °Brix.

The highest fructose content was recorded in haskap berry sorbet, at 7.25 g/100 g (Table 1c). Apple sorbet contained 4.17 g/100 g, while the remaining products contained between 1.40 and 2.75 g/100 g. Plum sorbet had the lowest fructose content. Haskap berry sorbet also had the highest glucose content, at 15.58 g/100 g. A high value was recorded for strawberry sorbet, at 9.83 g/100 g, followed by plum sorbet, at 6.07 g/100 g. Apple sorbet contained the least glucose, at 1.27 g/100 g. Sucrose content was highest in plum sorbet, at 20.26 g/100 g, followed by blackcurrant A and raspberry sorbets, at 19.65 and 19.18 g/100 g, respectively. In blackcurrant B, sucrose was below the detection limit, which was consistent with the formulation in which erythritol was used instead of sucrose. The sugar profile indicates that soluble solids content did not reflect only the amount of added sucrose. Haskap berry sorbet, despite its relatively low sucrose content, had the highest soluble solids value because of its high glucose and fructose contents and the presence of other soluble compounds. In contrast, plum sorbet had the highest sucrose content, but its soluble solids value was significantly lower than those of haskap berry and strawberry sorbets. The results showed a strong tendency for soluble solids and glucose content to vary together. Formulations with high glucose levels, particularly haskap berry and strawberry sorbets, also showed high soluble solids values. Nevertheless, refractometric °Brix measurements are influenced by other dissolved substances and therefore cannot be directly equated with the sum of the quantified sugars.

The heat of combustion of the dry matter varied within a relatively narrow range, from 15.95 to 17.38 kJ/g dry matter (Table 1c). The highest value was recorded for blackcurrant B, at 17.38 kJ/g, followed by raspberry sorbet, at 17.10 kJ/g. The lowest values were obtained for blackcurrant A and haskap berry sorbets, at 15.95 and 15.97 kJ/g dry matter, respectively.

The L* parameter, describing lightness, ranged from 13.47 to 48.90 (Table 1d). Apple sorbet was the lightest product, at 48.90, followed by plum sorbet, at 47.50. Both products belonged to the same statistical group. Blackcurrant B, haskap berry, and blackcurrant A sorbets were the darkest, with L* values of 13.47, 13.92, and 16.37, respectively. These formulations did not differ significantly in lightness. The pattern of lightness corresponded to anthocyanin content. Haskap berry and blackcurrant sorbets contained by far the highest amounts of anthocyanins and were also the darkest products. Apple and plum sorbets contained substantially fewer anthocyanins and showed the highest L* values. Exploratory analysis of the mean values indicated a very strong negative relationship between anthocyanin content and lightness. This confirms the dominant influence of anthocyanins on the visual intensity of dark-colored fruit sorbets. Positive a* values indicated a contribution of red color in all products. Raspberry sorbet showed the highest a* value, at 38.04, followed by strawberry sorbet, at 34.21 (Table 1d). The two blackcurrant formulations had similar values of 29.85 and 28.26. Haskap berry sorbet had a markedly lower red component, at 14.34. The lowest a* value was recorded for apple sorbet, at 4.38 ± 0.20. The result obtained for haskap berry indicates that, despite its very high anthocyanin content, it was not the reddest product. Its color was very dark and weakly saturated, probably with a greater contribution of purple or dark blue tones. This interpretation is supported by the low L*, b*, C*, and hue-angle values. The b* parameter was highest in apple sorbet, at 31.16, indicating a strong yellow component (Table 1d). High values were also recorded in plum and strawberry sorbets, at 27.46 and 24.70, respectively. The lowest b* value was observed for haskap berry sorbet, at 5.07. The two blackcurrant formulations had similar b* values of 13.18 and 11.07. Raspberry and strawberry sorbets showed the highest chroma values, C*, at 42.75 and 42.21, respectively (Table 1d). These products therefore exhibited intense and vivid red-orange colors. Haskap berry sorbet showed the lowest chroma, at 15.21. Thus, despite its high pigment concentration, its color was very dark but less vivid and less saturated than that of raspberry and strawberry sorbets. The two blackcurrant formulations did not differ significantly in L*, a*, b*, or C*. This indicates that replacing sucrose with erythritol did not substantially change the instrumentally measured lightness, red and yellow components, or color saturation. Their similar color profiles were also consistent with their identical anthocyanin contents.

The hue angle, *h*°, was highest in apple sorbet, at 82.00°, followed by plum sorbet, at 69.80° (Table 1d). These values indicate a shift towards yellow tones. Berry-based sorbets showed considerably lower hue angles, ranging from 19.37° for haskap berry to 35.77° for strawberry, corresponding to a greater red-color contribution. A clear tendency was observed whereby products with the highest antiradical activity showed the lowest hue-angle values. Haskap berry and blackcurrant sorbets, which contained high levels of anthocyanins and vitamin C and exhibited high antiradical activity, had colors shifted towards dark red and purple tones. Apple and plum sorbets, which showed lower antiradical activity, were characterized by more yellowish hues.

The ΔEa*b* calculated relative to the mean value of each formulation primarily represents within-product color variability or uniformity. It does not describe color differences between individual sorbet types. The ΔE*ab values calculated relative to the mean color of each formulation ranged from 0.50 to 2.72 and did not differ significantly among the sorbets (Table 1d). Apple sorbet showed the lowest within-formulation color variability, at 0.50, whereas haskap berry sorbet showed the highest, at 2.72. However, the absence of significant differences indicates that all products exhibited comparable color uniformity within the analyzed measurements.

### 2.2. Probiotic Properties of the Sorbets

The probiotic value of the sorbets was evaluated by several factors: the amount of *Lactiplantibacillus plantarum* 299v before and after freezing in fruit sorbets (log CFU/g). On the basis of these results the effects of the dose, sorbet type, and selected physicochemical properties of the matrix on the probiotic value of the sorbets were analyzed.

The results of *Lactiplantibacillus plantarum* 299v viability before and after freezing are given in Table 2 as the log CFU/g before and after freezing (sorbet mixture and final product), as well as log reduction and apparent survival (%).

The viability of *Lactiplantibacillus plantarum* 299v was evaluated in seven fruit sorbet matrices containing either one or two probiotic capsules. Before freezing, viable counts ranged from 7.01 to 7.19 log CFU/g in sorbets supplemented with one capsule and from 7.51 to 7.70 log CFU/g in products containing two capsules. After freezing, the corresponding values ranged from 7.05 to 7.18 log CFU/g and from 7.45 to 7.51 log CFU/g, respectively. All sorbet formulations retained viable counts above 7.0 log CFU/g after freezing. Thus, regardless of the fruit matrix and probiotic dose, the products maintained a high concentration of viable *Lactiplantibacillus plantarum* 299v cells. From a technological perspective, this indicates that the investigated sorbet matrices provided generally favorable protection of the strain during freezing. The response to freezing differed considerably between products containing one and two capsules. In the one-capsule variants, the mean viable count decreased only slightly, from approximately 7.12 to 7.11 log CFU/g. The mean log reduction was 0.01 log CFU/g, corresponding to a mean calculated survival of approximately 98.10%. The difference between counts before and after freezing was not statistically significant in the exploratory paired comparison across the seven fruit matrices.

Among the one-capsule formulations, strawberry sorbet showed the highest stability during freezing. Its viable count remained practically unchanged, decreasing by only 0.001 log CFU/g, which corresponded to 99.85% survival. Apple and haskap berry sorbets also showed very low reductions of 0.01 and 0.02 log CFU/g, respectively, with survival values of 98.05 and 95.41%. Blackcurrant A and raspberry sorbets showed slightly greater reductions of 0.05 and 0.06 log CFU/g, corresponding to 90.26 and 87.47% survival, respectively. However, even in these products, the loss of viability was small.

In blackcurrant B and plum sorbets containing one capsule, viable counts after freezing were slightly higher than before freezing. The calculated log reductions were −0.04 and −0.02 log CFU/g, while apparent survival exceeded 100%, reaching 109.65 and 105.85%, respectively. Such values should not be interpreted as actual multiplication of bacteria during freezing. They most probably reflect normal analytical variability associated with microbiological enumeration, sampling heterogeneity, cell-cluster dispersion, or improved release of bacterial cells from the matrix during sample preparation after freezing.

The two-capsule formulations displayed a different ranking. Haskap berry sorbet showed the lowest reduction, at 0.06 log CFU/g, and the highest survival, at 87.50%. Blackcurrant B was the second most protective matrix, with a reduction of 0.08 log CFU/g and survival of 82.41%. Strawberry sorbet showed a reduction of 0.11 log CFU/g and survival of 79.53%. The largest decrease was observed in apple sorbet containing two capsules. Its viable count declined by 0.19 log CFU/g, corresponding to only 63.97% survival. Raspberry, blackcurrant A, and plum sorbets showed identical reported reductions of 0.15 log CFU/g and identical survival values of 71.82%.

These results indicate that the protective effect of the fruit matrix depended partly on probiotic dose. A matrix that performed well at the lower bacterial concentration did not necessarily provide equally effective protection when the initial cell load was increased. Apple sorbet was a clear example: it showed almost complete survival in the one-capsule formulation but the lowest survival in the two-capsule formulation.

The type of sorbet and the capsule number added had a significant effect on the number of *Lactiplantibacillus plantarum* 299v (Table 3a–e). The mixed-effects ANOVA showed that viable cell counts differed according to sorbet type, capsule number, and freezing condition (Table 3a). The influence of capsule number varied both among sorbet formulations and between measurements taken before and after freezing, as indicated by the significant sorbet × capsule-number and capsule-number × freezing interactions. No evidence was found that the freezing response differed among sorbets or that it depended jointly on sorbet type and capsule number.

Planned before–after contrasts showed that freezing did not measurably alter viable counts in samples containing one capsule (estimated difference $=0.0105{log}_{10}$ CFU/g, p=0.4578). In the two-capsule condition, however, the post-freezing count was $0.1267{log}_{10}$ CFU/g lower than the initial value, which was marked as a significant difference in a *t*-test (p<0.0001) (Table 3b).

Comparison of the two capsule-number conditions demonstrated higher viable counts in formulations containing two capsules at both measurement points (Table 3c). The difference amounted to $0.489{log}_{10}$ CFU/g before freezing and $0.373{log}_{10}$ CFU/g after freezing, with both contrasts reaching statistical significance (p<0.0001). The smaller post-freezing advantage corresponds to the significant capsule-number × freezing interaction.

Analysis of freezing-related log reduction revealed no overall differences among sorbet types, whereas capsule number had a significant effect (Table 3d).

The significant sorbet × capsule-number interaction further indicated that the magnitude of this effect depended on the formulation. On average, log reduction was $0.116{log}_{10}$ units lower with one capsule than with two capsules (p<0.0001), corresponding to a higher proportional survival rate in the one-capsule condition (Table 3e).

The results confirm that freezing did not significantly reduce viability in the one-capsule formulations. In contrast, a significant decrease was observed when two capsules were added. Nevertheless, the two-capsule products still retained significantly higher absolute counts after freezing.

### 2.3. The Influence of Physicochemical Properties on Viability of Lactiplantibacillus plantarum 299v

**Figure 2:**
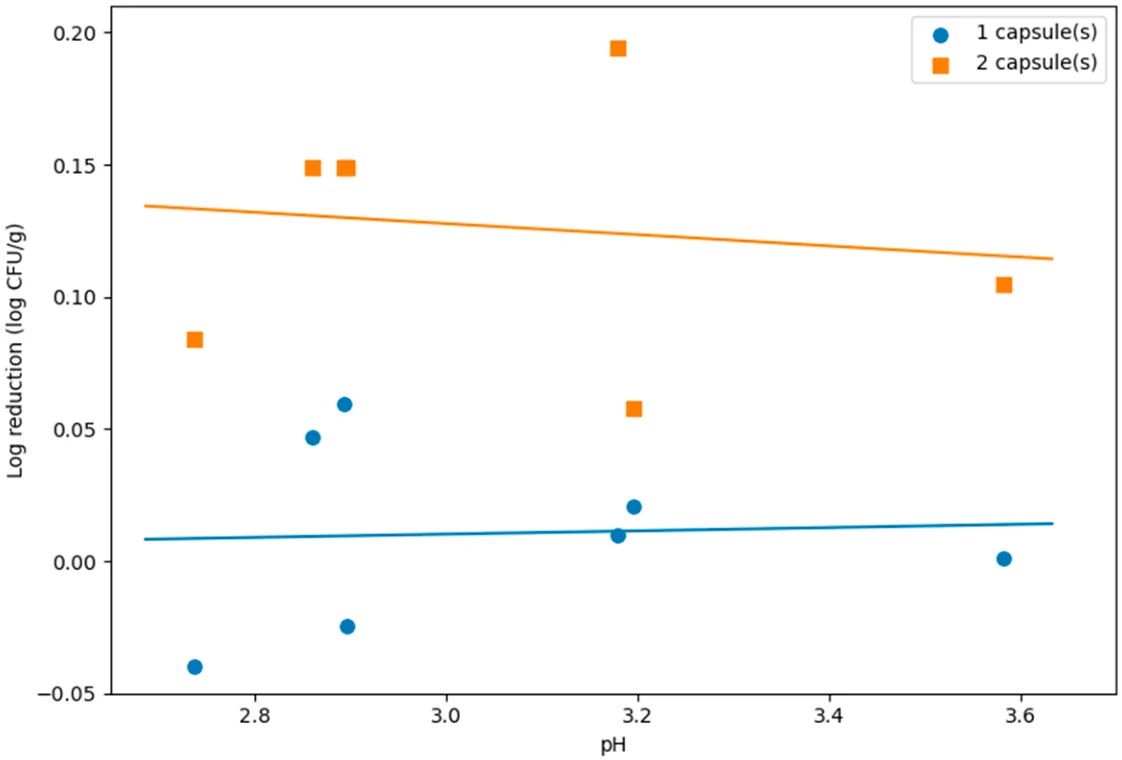
Exploratory pH–log reduction relationship.

Many factors can influence the viability of *Lactiplantibacillus plantarum* 299v; one of the most important is the acidity of the matrix. The pH values of the sorbets ranged from 2.74 to 3.58. Despite this strongly acidic environment, *Lactiplantibacillus plantarum* 299v remained highly viable in all matrices (Figure 2). This confirms the good acid tolerance of the strain and its suitability for incorporation into acidic fruit-based frozen desserts. No simple linear relationship was observed between pH and survival after freezing. Strawberry sorbet, which had the highest pH of 3.58, showed almost complete survival in the one-capsule formulation and relatively high survival in the two-capsule formulation. However, blackcurrant B, which had the lowest pH of 2.74, also provided very good protection, particularly in comparison with other two-capsule sorbets. Similarly, plum sorbet had a low pH of 2.90 but showed an apparent survival above 100% with one capsule. These findings indicate that low pH alone was not the dominant factor determining freezing resistance. The absence of a clear adverse relationship may be explained by the intrinsic acid adaptation of *Lactiplantibacillus plantarum*. Exposure to an acidic fruit matrix before freezing might also induce stress-response mechanisms that could provide cross-protection against subsequent cold and freezing stress.

Potential acidity varied considerably among the sorbets, from 2.76°SH in strawberry sorbet to 9.43°SH in haskap berry sorbet. As with pH, no direct negative effect of higher titratable acidity was evident. Haskap berry sorbet, despite having by far the highest potential acidity, showed high survival at both probiotic doses. It retained 95.41% of viable cells in the one-capsule variant and 87.50% in the two-capsule variant, the latter being the highest survival value among all two-capsule formulations. This result suggests that high total acidity did not necessarily impair *Lactiplantibacillus plantarum* 299v viability. The protective effects of other matrix components in haskap berry sorbet may have outweighed the potential stress associated with its high organic acid content.

No significant relationship was found between sorbet pH and the reduction in *Lactiplantibacillus plantarum* 299v viability during freezing (Table 4). For formulations containing one capsule, both the Pearson correlation (r = 0.051, *p* = 0.913) and Spearman correlation (ρ = 0.036, *p* = 0.939) were close to zero. Similarly, no association was observed in sorbets containing two capsules, as indicated by negligible Pearson and Spearman coefficients and non-significant *p*-values (*p* = 0.778 and *p* = 0.812, respectively). These results indicate that differences in pH among the investigated sorbet matrices did not explain the variation in log reduction after freezing. Thus, within the analyzed pH range, acidity expressed as pH was not a major determinant of *L. plantarum* 299v survival.

The distinction between pH and potential acidity is also important. pH reflects the concentration of free hydrogen ions, whereas titratable acidity represents the overall acid reservoir and buffering capacity of the product. A highly buffered matrix may expose cells to acid stress but may also reduce rapid local fluctuations in pH during freezing and thawing. Therefore, the effect of acidity on probiotic survival is likely to depend on the composition of organic acids and the buffering properties of the sorbet rather than on a single acidity parameter.

Sugars can protect bacterial cells during freezing by reducing ice formation, lowering the freezing point, retaining water in the unfrozen phase, and stabilizing cell membranes and proteins [51,52,53]. The present study indicates that the protective effect depended on the type and combination of sugars rather than simply on their total concentration. Haskap berry sorbet contained the highest soluble solids content, at 35.80 °Brix, and the highest glucose and fructose concentrations. It also showed the best survival among the two-capsule formulations. This suggests that its high concentration of soluble compounds may have provided effective cryoprotection, particularly when the probiotic load was high. Glucose content showed a relatively strong negative exploratory relationship with log reduction in the two-capsule products. In other words, matrices containing more glucose tended to exhibit smaller viability losses. Haskap berry sorbet, which contained 15.58 g/100 g glucose, had the lowest reduction, whereas apple sorbet, which contained only 1.27 g/100 g glucose, had the largest reduction. This trend suggests that glucose and the overall soluble phase may have contributed to maintaining a larger fraction of unfrozen water and limiting mechanical damage caused by ice crystals. Nevertheless, the small number of sorbet types means that this relationship should be interpreted cautiously. Fructose did not show an equally clear independent association with survival. Although haskap berry sorbet contained the highest fructose concentration and showed good viability, apple sorbet also contained relatively high fructose but had the poorest survival in the two-capsule group. Therefore, fructose alone cannot explain the observed differences. Sucrose content also did not show a consistently protective effect. Raspberry, blackcurrant A, and plum sorbets contained approximately 19–20 g/100 g sucrose but displayed relatively large reductions in the two-capsule group. In contrast, blackcurrant B contained no detectable sucrose and showed the second-highest survival among the two-capsule formulations.

The comparison between blackcurrant A and blackcurrant B is especially informative. Both products were based on the same fruit and had very similar pH, but differed in sugar content. Blackcurrant B, in which sucrose was replaced by erythritol, showed better survival after freezing at both probiotic doses. In the two-capsule variants, the log reduction was 0.084 for blackcurrant B compared with 0.149 for blackcurrant A. This difference suggests that erythritol may have contributed to cryoprotection or that the modified physical structure of blackcurrant B improved cell preservation. Polyols can interact with water and alter ice crystallization [54,55].

Total solids content ranged from 25.52% in blackcurrant B to 34.53% in haskap berry sorbet. In general, higher total solids can enhance probiotic survival by reducing the proportion of freezable water and increasing the viscosity of the continuous phase. However, total solids alone did not determine viability in this study.

Overrun may affect probiotic survival by modifying product density, ice-crystal distribution, oxygen incorporation, and the physical location of bacterial cells within the matrix [56,57,58]. In the exploratory analysis of two-capsule formulations, higher overrun tended to be associated with greater log reduction. A more highly aerated product may expose bacterial cells to greater interfacial stress and oxygen. Air incorporation can also produce thinner regions of the unfrozen matrix between ice crystals and air cells, potentially increasing mechanical or osmotic stress [59,60]. Nevertheless, this trend was not statistically conclusive and should be regarded as a hypothesis requiring direct verification. The relationship observed in the one-capsule formulations was less consistent. At the lower probiotic concentration, viability was generally very high, and the small variation in log reduction may have been dominated by analytical variability rather than matrix-related effects.

Bioactive compounds may indirectly support viability by limiting oxidative damage during processing and frozen storage. Freezing can disturb cell membranes and increase bacterial susceptibility to reactive oxygen species during thawing. A fruit matrix with high reducing capacity may therefore provide additional protection [61,62,63,64].

Haskap berry and blackcurrant sorbets contained the highest levels of anthocyanins, vitamin C, and antiradical activity. These matrices generally supported good survival, particularly in the two-capsule group. Exploratory correlations indicated that higher vitamin C content, antiradical activity, and anthocyanin content tended to be associated with smaller log reductions in the two-capsule formulations. The relationship was strongest for vitamin C, although it was based on only seven observations. However, phenolic compounds may also exert antimicrobial effects, depending on their structure and concentration. The high survival observed in anthocyanin-rich sorbets suggests that, under the conditions of this study, the antioxidant or matrix-protective effects did not produce a measurable inhibition of *Lactiplantibacillus plantarum* 299v. Total polyphenol content alone did not clearly predict survival. Plum sorbet had the highest polyphenol concentration but did not show particularly good preservation in the two-capsule formulation. As observed for antiradical activity, the qualitative profile of phenolic compounds may be more relevant than their total amount.

### 2.4. Consumer Evaluation of the Sorbets

Consumer evaluation revealed significant differences among the seven sorbet formulations for all analyzed sensory attributes. Repeated-measures ANOVA showed a significant effect of sorbet type on general appearance, color, taste, odor, consistency, structure, and overall acceptance, with *p* < 0.001 for every attribute (Table 5). The largest differentiation between formulations was observed for color (*F* = 35.42), followed by general appearance (*F* = 26.48), overall acceptance (*F* = 25.65), structure (*F* = 23.93), consistency (*F* = 22.00), taste (*F* = 17.25), and odor (*F* = 15.53). The largest between-product differentiation was observed for color, followed by general appearance and overall acceptance. Odor had the lowest *F*-value, although the effect of sorbet type remained highly significant.

These results demonstrate that the type of fruit matrix and the formulation of the sorbet had a substantial effect on consumer perception. The particularly high *F*-value obtained for color indicates that visual differences between the products were especially pronounced and consistently perceived by the panel. At the same time, the significant results for taste, texture-related attributes, and overall acceptance show that consumer preferences were shaped by a combination of visual, flavor, and structural properties rather than by appearance alone. Because the same 117 consumers evaluated all seven sorbets, consumer was included as a blocking factor. For each sensory attribute, a repeated-measures/randomized complete block ANOVA was fitted, followed by Tukey-adjusted pairwise comparisons based on the residual mean square of the blocked model. Mean consumer acceptance scores are presented in Table 6.

Overall acceptance scores ranged from 5.86 to 7.78 points. The highest mean value was recorded for blackcurrant B sorbet, at 7.78, followed by raspberry sorbet at 7.62 and strawberry sorbet at 7.57. Blackcurrant A also received a relatively high score of 7.25. These four products belonged to the highest homogeneous group and did not differ significantly from one another in overall acceptance. Plum sorbet received an intermediate overall acceptance score of 6.54. Haskap berry sorbet obtained a similar mean score of 6.42 and shared statistical-group membership with both plum and apple sorbets. Apple sorbet received the lowest overall acceptance score, at 5.86. The results therefore identify two broad consumer-preference profiles. Raspberry, strawberry, and both blackcurrant formulations were highly accepted, whereas haskap berry, plum, and particularly apple sorbets received lower ratings. However, the overlapping Tukey groups indicate that the boundaries between the intermediate and least accepted products were not absolute. Haskap berry sorbet, for example, did not differ significantly from either plum or apple sorbet, although plum and apple sorbets were assigned to different homogeneous groups. Blackcurrant B was particularly noteworthy because it achieved the numerically highest overall acceptance despite the replacement of sucrose with erythritol. This indicates that the reformulation did not impair consumer acceptance and may even have improved the sensory profile relative to blackcurrant A. However, both blackcurrant formulations belonged to the same Tukey group for overall acceptance; the numerical difference is statistically insignificant.

General appearance scores ranged from 6.05 to 7.86 points. Strawberry sorbet received the highest mean rating, at 7.86, followed closely by raspberry at 7.76, blackcurrant B at 7.71, and blackcurrant A at 7.45. These formulations formed a common homogeneous group. Haskap berry, plum, and apple sorbets obtained significantly lower scores, at 6.50, 6.62, and 6.05, respectively. These three products belonged to the same lower statistical group. The results indicate that consumers preferred the appearance of raspberry, strawberry, and blackcurrant sorbets. These products were characterized by more intense red or dark-red coloration, whereas apple and plum sorbets were considerably lighter and more yellow. Haskap berry sorbet, although rich in anthocyanins, was very dark and showed low color saturation, which may have reduced its visual attractiveness. The relatively large standard deviation observed for plum sorbet suggests greater variation in individual responses. Some consumers may have found its appearance attractive, whereas others rated it considerably less favorably. In contrast, blackcurrant B had a smaller standard deviation, indicating more consistent acceptance of its appearance.

Color was the attribute most strongly differentiated by sorbet type. Ratings ranged from 5.93 to 8.05 points. Blackcurrant B obtained the highest numerical score, at 8.05, followed by strawberry at 7.97, raspberry at 7.77, and blackcurrant A at 7.73. These four formulations belonged to the same highest statistical group. Haskap berry sorbet received an intermediate color score of 6.98 and differed significantly from the products in the highest group. Plum and apple sorbets received the lowest ratings, at 6.29 and 5.93, respectively, and did not differ significantly from one another.

The consumer results were consistent with the instrumental color measurements. Raspberry and strawberry sorbets had the highest chroma values and strong positive a* values, indicating vivid and saturated red color. Both formulations received high consumer ratings for color. Blackcurrant sorbets were considerably darker but retained a strong red component and also received very high color scores. In contrast, apple and plum sorbets had the highest L* and hue-angle values, corresponding to lighter and more yellow coloration. These products received the lowest consumer color ratings. The exploratory comparison of mean values indicated a strong positive relationship between the instrumental a* parameter and overall acceptance. Products with a stronger red component tended to be more highly accepted. A negative tendency was observed between hue angle and overall acceptance. Sorbets with higher hue angles, representing a shift towards yellow tones, tended to receive lower acceptance scores. This pattern suggests that consumers preferred red, dark-red, or berry-like colors over pale yellow or yellow-brown tones in the investigated sorbet category. The result for haskap berry demonstrates that high pigment concentration alone did not guarantee high color acceptance. Although haskap berry sorbet had the highest anthocyanin content, it also had the lowest chroma and one of the lowest L* values. Its color was therefore very dark but comparatively dull or weakly saturated. Consumers may have preferred the brighter, more vivid appearance of raspberry, strawberry, and blackcurrant sorbets.

Taste scores ranged from 6.25 to 7.98 points. Blackcurrant B received the highest mean rating, at 7.98, followed by raspberry at 7.60 and strawberry at 7.56. Blackcurrant A received a score of 7.20, while plum sorbet was rated at 6.73. Haskap berry and apple sorbets obtained the lowest numerical scores, at 6.37 and 6.25, respectively. Blackcurrant B was among the best-rated formulations and was significantly preferred over several lower-scoring products. Raspberry and strawberry sorbets also received high taste ratings, although their overlapping group membership indicates partial similarity with some intermediate formulations. The high taste acceptance of blackcurrant B is particularly important because this formulation contained erythritol instead of sucrose. The result indicates that replacing sucrose did not produce an unacceptable sweetness profile or an excessively strong cooling or off-flavor sensation. On the contrary, blackcurrant B achieved the highest numerical taste score and exceeded blackcurrant A by 0.78 points. Several factors may have contributed to this result. Blackcurrant B had the lowest pH but a potential acidity similar to blackcurrant A. It therefore retained the characteristic tartness of blackcurrant, while erythritol may have altered the balance between sweetness and acidity. Its lower energy value and absence of detectable sucrose did not compromise taste acceptance. Haskap berry sorbet showed the highest potential acidity and high concentrations of glucose and fructose, but its taste acceptance was relatively low. This suggests that natural sugar concentration did not fully compensate for the intense acidity or characteristic flavor profile of the fruit. The high total soluble solids content of this formulation therefore did not translate directly into higher consumer liking. Apple sorbet also received a low taste rating despite having moderate pH and relatively high energy value. Its low antioxidant activity and weak red color were unlikely to affect taste directly, but its sugar–acid balance and comparatively mild fruit character may have been less attractive than the more pronounced flavors of raspberry, strawberry, and blackcurrant. Plum sorbet had the highest sucrose content but only intermediate taste acceptance. This observation confirms that sucrose concentration alone was not the principal determinant of taste preference. Consumer liking was more likely influenced by the balance among sweetness, acidity, fruit aroma, and possible astringent or bitter notes.

Odor ratings ranged from 6.21 to 7.72 points. Strawberry sorbet achieved the highest score, at 7.72, followed by blackcurrant B at 7.44 and raspberry at 7.41. These formulations formed the highest statistical group. Blackcurrant A, plum, haskap berry, and apple sorbets received lower ratings, ranging from 6.21 to 6.63, and belonged to the lower homogeneous group. Apple sorbet obtained the lowest mean odor score, at 6.21. The high rating of strawberry sorbet may be associated with its characteristic and readily recognizable aroma, which is generally strongly linked with consumer expectations for fruit desserts. Raspberry also possesses an intense and familiar aroma profile, contributing to its high score. The difference between blackcurrant A and blackcurrant B was notable. Blackcurrant B received an odor rating of 7.44, compared with 6.60 for blackcurrant A, and the formulations were assigned to different statistical groups. Because the fruit base was the same, the difference may have resulted from the sweetener system or interactions between volatile compounds and the frozen matrix. Replacing sucrose with erythritol may have altered the release or perception of aroma compounds. Differences in total solids, overrun, and product structure could also have influenced volatile release during consumption. Blackcurrant B had considerably lower overrun and greater hardness, potentially modifying the rate at which aroma compounds were released as the product melted in the mouth.

Consistency ratings ranged from 5.79 to 7.62 points. Blackcurrant B received the highest numerical score, at 7.62, followed by raspberry at 7.56, blackcurrant A at 7.47, strawberry at 7.40, and plum at 6.68. Raspberry, strawberry, and both blackcurrant formulations belonged to the highest homogeneous group. Plum sorbet received an intermediate score, while haskap berry and apple sorbets were rated lower, at 6.38 and 5.79, respectively. Apple sorbet had the lowest consistency score. These results are relevant in relation to instrumental hardness. Blackcurrant B was the hardest product but received the highest consistency rating. Apple sorbet was the softest product and obtained the lowest consistency score. This suggests that, within the investigated formulations, consumers preferred a firmer and more compact consistency rather than a very soft product.

The exploratory relationship between hardness and overall acceptance was positive. However, this should not be interpreted as evidence that increasing hardness will always improve consumer liking. The observed association was strongly influenced by blackcurrant B, which combined high hardness with excellent ratings, and apple sorbet, which combined low hardness with poor ratings. The preferred consistency was likely determined by an optimal balance among firmness, smoothness, melting rate, and resistance to deformation. Excessive softness may have been perceived as watery or insufficiently frozen, whereas a firmer structure may have been associated with greater body and product quality. Haskap berry sorbet had intermediate hardness but a low consistency rating. This indicates that hardness alone did not determine consumer perception. Its high total solids content, low overrun, and distinct fruit composition may have produced a dense or less smooth mouthfeel, thereby reducing acceptability.

Structure scores ranged from 5.71 to 7.50 points. Raspberry sorbet received the highest mean rating, at 7.50, followed by strawberry at 7.48, blackcurrant B at 7.45, and blackcurrant A at 7.27. These four formulations belonged to the highest homogeneous group. Plum sorbet received an intermediate score of 6.63. Haskap berry sorbet was rated at 6.24, while apple sorbet received the lowest score, at 5.71. Structure and consistency showed almost identical patterns of consumer preference. This indicates that consumers perceived the physical properties of the sorbets consistently across related sensory attributes. Products rated as having a favorable consistency were generally also considered to have a desirable structure. Raspberry and strawberry sorbets combined high overrun with high structure scores. Their greater incorporation of air may have produced a lighter and smoother structure. Blackcurrant B, despite having the lowest overrun, was also highly rated, suggesting that a different structural profile—more compact and firm—could also be acceptable. Thus, there was no single overrun level that determined consumer preference. Both highly aerated berry sorbets and the minimally aerated blackcurrant B formulation were well accepted. The effect of overrun depended on interactions with hardness, ice-crystal distribution, total solids, and sweetener composition. Apple sorbet combined high overrun with the lowest hardness and the lowest structure score. This suggests that its structure may have been perceived as excessively soft, weak, or insufficiently cohesive. Conversely, the lower structure rating of haskap berry sorbet may have been related to its dense composition, high total solids, and relatively low overrun.

All sensory attributes were strongly related to overall acceptance (Table 7). Exploratory correlations calculated from the seven formulation means showed that all sensory attributes were strongly positively associated with overall acceptance. General appearance showed the strongest association, followed by structure, consistency, and taste. These results indicate that consumer liking reflected a combined response to visual quality, flavor, and frozen-product texture.

The strongest relationship was observed for general appearance, with a correlation coefficient of approximately r = 0.99. Structure and consistency were also very strongly related to overall acceptance, with coefficients of approximately r = 0.98. Taste showed a similarly strong relationship with overall acceptance, at approximately r = 0.98. Color was also strongly associated with overall liking, at approximately r = 0.95, while odor showed a somewhat weaker but still substantial relationship, at approximately r = 0.89. These results indicate that overall acceptance was a multidimensional response. Although taste is commonly considered the principal determinant of food acceptance, in the present study visual appearance and texture-related properties were at least equally strongly associated with the overall rating. The very strong relationship between general appearance and overall acceptance may partly reflect the initial influence of visual perception on subsequent product evaluation. A visually attractive sorbet may create positive expectations regarding flavor and quality before tasting. The strong relationships between consistency, structure, and overall acceptance demonstrate the importance of frozen-product texture. Consumers appeared to prefer products with a clearly defined, cohesive, and appropriately firm structure. The low acceptance of apple sorbet was associated not only with its lower taste and color ratings but also with its particularly low consistency and structure scores.

### 2.5. Differences in Sensory Attributes Among Sorbet Formulations

Differences in sensory attributes among seven sorbet formulations were analyzed using the Friedman repeated-measures test. Kendall’s W was reported as a measure of effect size and consumer concordance. For all attributes, *p* < 0.001. According to the Friedman test and Kendall’s W values product differences were significant for all attributes (Table 8).

The Friedman test results confirm that the fruit matrix and the sorbet significantly affected all evaluated sensory attributes. Statistically significant differences among the seven formulations were found for general appearance, color, taste, odor, consistency, structure, and overall acceptance, with *p* < 0.001 in every case. Thus, the null hypothesis that consumers evaluated all sorbet formulations similarly was rejected for each sensory attribute.

The strongest differentiation among the products was observed for color, which was the sensory characteristic most strongly influenced by the type of fruit matrix and for which consumers showed the greatest consistency in distinguishing the formulations according to this attribute. The result agrees with the pronounced instrumental differences in lightness, redness, yellowness, chroma, and hue observed among the sorbets. The sensory attributes can be ranked according to the magnitude of the formulation effect as follows: color (*W* = 0.243), overall acceptance (*W* = 0.197), general appearance (*W* = 0.189), structure (*W* = 0.181), consistency (*W* = 0.166), taste (*W* = 0.143), and Odor (*W* = 0.136). This ranking indicates that visual characteristics were the most discriminating sensory dimension, followed by overall liking and texture-related properties. Taste and odor, although statistically significant, showed greater interindividual variability.

### 2.6. Relationships Between Individual Sensory Attributes and Overall Acceptance

In sensory analysis and consumer preference studies, the relationship between individual sensory attributes and overall acceptance is of great importance. Therefore such an analysis using Spearman correlations was performed. The sensory attributes were correlated with one another. Although the results do not prove that changing a single attribute would independently increase overall acceptance, the consistently strong coefficients indicate that improvements in taste, structure, and consistency would be particularly relevant when reformulating the less accepted sorbets.

Spearman correlation analysis showed that all evaluated sensory attributes were strongly and positively associated with overall acceptance (Table 9). All correlations were statistically significant at *p* < 0.001, indicating that higher ratings of appearance, color, taste, odor, consistency, and structure were consistently accompanied by higher overall liking. The strongest relationship was observed for taste, with Spearman’s ρ = 0.859. This indicates that taste was the sensory attribute most closely associated with consumers’ overall acceptance of the sorbets. Products receiving higher taste scores were therefore very likely to receive higher overall ratings. The strength of this association confirms that flavor quality, including sweetness–acidity balance and the characteristic fruit profile, was a major determinant of product acceptance. The strength of the relationships can be ranked as follows: taste (ρ = 0.859), structure (ρ = 0.823), consistency (ρ = 0.817), color (ρ = 0.752), odor (ρ = 0.749), and general appearance (ρ = 0.738). This ranking indicates that overall acceptance was most strongly associated with taste and texture-related properties, whereas visual and aromatic characteristics, although also important, showed slightly weaker relationships.

These findings complement the Friedman test results. The Friedman analysis showed which attributes most strongly differentiated the sorbet formulations, whereas the Spearman correlations indicate which sensory scores were most closely related to overall acceptance. Color produced the strongest differentiation among products, but taste showed the strongest relationship with overall liking. Therefore, an attribute may be highly effective in distinguishing products without necessarily being the principal driver of acceptance.

Overall, the results demonstrate that consumer acceptance of the sorbets was multidimensional. Taste appeared to be the most important correlate of overall liking, but structure and consistency were nearly as influential. Color, odor, and general appearance also made substantial contributions. Consequently, successful product optimization should not focus exclusively on flavor; it should also ensure an attractive color and a desirable frozen-dessert texture.

### 2.7. Comparison of the Blackcurrant Formulations

One of the aims of the study was the comparison of consumer evaluation of two blackcurrant matrices of the sorbets. The comparison between blackcurrant A and blackcurrant B is particularly relevant for product development because the formulations differed mainly in the sweetener system. The consumer scores and differences in those scores are presented in Table 10. Blackcurrant B received numerically higher ratings for every attribute. The clearest statistically supported difference concerned odor, because blackcurrant A and blackcurrant B belonged to different homogeneous groups. For the remaining attributes, the formulations shared or overlapped in Tukey group membership, so numerical differences are statistically insignificant.

Blackcurrant B received higher numerical ratings for every sensory attribute. The differences between blackcurrant B and blackcurrant A amounted to 0.26 points for general appearance, 0.32 for color, 0.78 for taste, 0.84 for odor, 0.15 for consistency, 0.18 for structure, and 0.53 for overall acceptance. The largest differences were observed for odor and taste. According to the Tukey-adjusted comparisons, the odor ratings of the two blackcurrant formulations belonged to different homogeneous groups (Table 6), indicating a significant difference. For several other attributes, including overall acceptance, the formulations shared group membership; therefore, their numerical differences should not be regarded as statistically significant. Nevertheless, the consistent direction of all differences suggests that replacing sucrose with erythritol did not reduce sensory quality. Blackcurrant B also had a lower energy value, no detectable sucrose, high probiotic viability, and retained antioxidant and color characteristics comparable with blackcurrant A. Blackcurrant B was instrumentally harder and less aerated than blackcurrant A, but these changes were not perceived negatively. On the contrary, blackcurrant B received the highest consistency score and a high structure rating. This suggests that the more compact structure produced by erythritol was acceptable or even desirable to consumers.

### 2.8. Purchase Intention and Its Relationship with Consumer Acceptance

Purchase intention was measured on a five-category ordinal scale coded from 1 (No) to 5 (Yes). Positive purchase intention was defined as the combined proportion of “Probably yes” and “Yes” responses. Valid response counts varied from 112 to 117 depending on the product (Table 11). The omnibus repeated-measures comparison was based on 98 consumers with complete valid purchase-intention responses for all seven sorbets.

Purchase intention differed significantly (Table 12) among the sorbet formulations (Friedman χ^2^(6) = 102.47, *p* < 0.001, Kendall’s W = 0.174). The effect size indicates a small-to-moderate but systematic differentiation of the products. Thus, although consumers did not show perfect agreement, formulation type materially influenced their declared willingness to buy.

The Friedman test result indicated that at least one sorbet differed from the others, but the test itself did not indicate which pairs of products differed significantly. This required the results of the Wilcoxon signed-rank tests for the sorbet pairs. Pairwise differences in purchase intention among seven sorbet formulations were assessed using two-sided Wilcoxon signed-rank tests based on paired consumer responses. Purchase intention was coded on a five-point ordinal scale: 1 = No, 2 = Probably no, 3 = Undecided, 4 = Probably yes, and 5 = Yes. *p*-values were adjusted for multiple comparisons using Holm’s method, and statistical significance was defined as Holm-adjusted *p* < 0.05. Raw *p*-values are also reported for reference. Mean values are shown only to facilitate interpretation of the direction of the differences; the Wilcoxon test itself is based on the ranks of paired within-consumer differences. The detailed results are given in Table S1 (Supplementary File).

Of the 21 pairwise comparisons, 16 yielded raw *p*-values below 0.05. After Holm adjustment, 13 comparisons remained statistically significant, while eight were not significant: raspberry versus strawberry, raspberry versus blackcurrant B, haskap berry versus plum, haskap berry versus apple, plum versus apple, raspberry versus blackcurrant A, plum versus blackcurrant A, and strawberry versus blackcurrant B (Table 13). The latter three comparisons were significant based on the raw *p*-values but did not remain significant after correction for multiple testing. These results indicate the presence of three broad purchase-intention tiers, although the overlapping non-significant pairwise differences mean that these tiers should be interpreted as general patterns rather than as strictly separated statistical groups.

Strawberry, raspberry, and blackcurrant B represented the highest purchase-intention group. No significant differences remained among these three formulations after Holm adjustment: raspberry did not differ from strawberry (Holm-adjusted *p* = 1.000) or blackcurrant B (*p* = 0.998), and the previously significant difference between strawberry and blackcurrant B was no longer significant after correction (*p* = 0.084). Blackcurrant A occupied an intermediate position, differing significantly from strawberry and blackcurrant B but not from raspberry or plum. Haskap berry, plum, and apple formed the lower purchase-intention group, with no significant pairwise differences among them. Overall, the Wilcoxon analysis showed clear differences in purchase intention among sorbet formulations, while the Holm-adjusted results also demonstrated overlap between adjacent groups. The direct superiority of blackcurrant B over blackcurrant A (Holm-adjusted *p* = 0.029) is particularly important for product development, because it demonstrates that the erythritol-sweetened formulation combined favorable sensory acceptance with a higher declared willingness to purchase.

**Figure 3:**
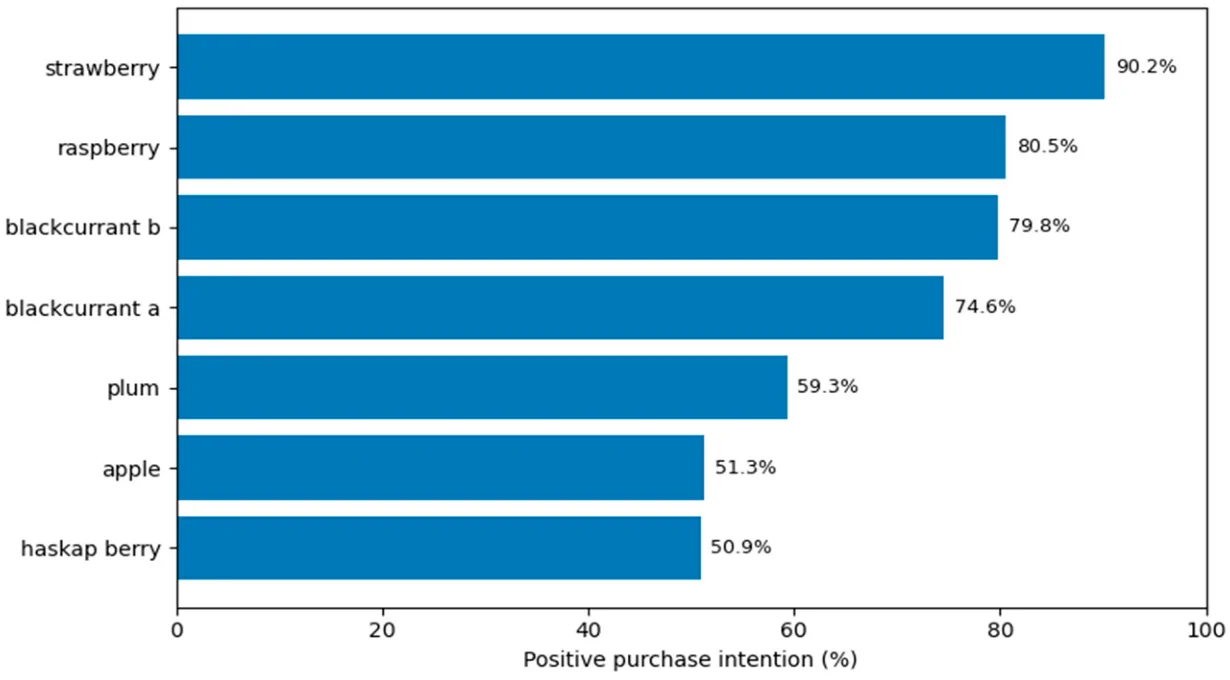
Percentage of positive purchase-intention responses (Probably yes + Yes).

Positive purchase-intention responses (Probably yes + Yes) were analyzed and the results are presented in Figure 3. Strawberry sorbet showed the highest mean purchase-intention score (4.36) and the largest proportion of positive responses (90.18%). It was the only product for which approximately nine in ten valid respondents selected either “Probably yes” or “Yes”. Raspberry ranked second in mean purchase intention (4.27) and reached 80.53% positive intention. Blackcurrant B also performed strongly, with a mean of 4.16 and 79.82% positive intention. Blackcurrant A achieved a lower but still favorable result: 74.56% of valid respondents declared positive purchase intention. Plum occupied an intermediate market position, with a mean score of 3.61 and 59.29% positive responses. Haskap berry and apple had the lowest positive purchase intention, at 50.89% and 51.28%, respectively. Their similar positive percentages concealed different response structures: haskap berry received slightly more definite “Yes” responses, whereas apple generated the largest undecided group.

Raspberry had the largest proportion of definite buyers: 60 of 113 valid respondents selected “Yes” (53.10%). Strawberry followed with 50.00%. Blackcurrant B and blackcurrant A reached 38.60% and 36.84%, respectively. Apple had the lowest definite-buying share, at 17.09%. The undecided fraction was particularly high for apple (28.21%) and haskap berry (25.89%), indicating that these formulations did not evoke uniformly negative reactions but frequently failed to generate a firm purchase decision. This distinction is relevant for optimization: undecided consumers may be more responsive to improvements in flavor, appearance, communication of functional benefits, price, or serving format than consumers who explicitly selected a negative category.

**Figure 4:**
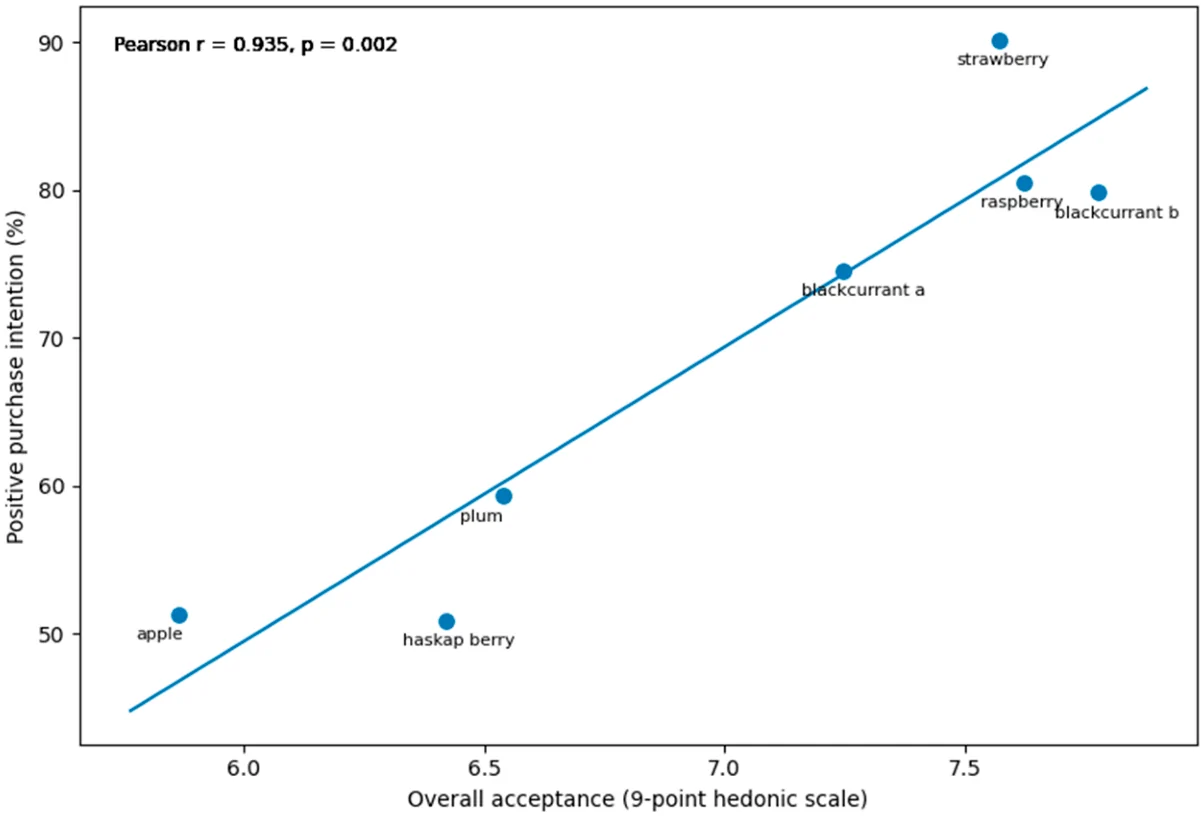
Product-level relationship between overall acceptance and positive purchase intention.

The impact of acceptance on purchase intentions and the relationship between overall acceptance and purchase intention indicators are extremely important. The results of the analysis of these parameters are given in Table 14 and Figure 4. At the formulation level, overall acceptance was very strongly and positively associated with purchase intention. The Pearson correlation between mean overall acceptance and the mean purchase-intention category was r = 0.948 (*p* = 0.001). The corresponding Spearman rank correlation was ρ = 0.857 (*p* = 0.014). A similarly strong relationship was obtained when purchase intention was expressed as the percentage of positive responses. The Pearson coefficient was r = 0.934 (*p* = 0.002), whereas the Spearman coefficient was ρ = 0.821 (*p* = 0.023). These results show that products with higher hedonic acceptance generally generated a stronger declared willingness to purchase.

The general alignment between acceptance and purchase intention was evident at both ends of the product ranking. Apple had the lowest overall acceptance (5.86) and one of the lowest positive purchase-intention percentages (51.28%). Haskap berry was also rated relatively poorly for overall acceptance (6.42) and had the lowest positive purchase intention (50.89%). These findings confirm that a strong functional and antioxidant profile does not automatically translate into market appeal. The relationship was not perfectly one-to-one. Blackcurrant B achieved the highest overall acceptance (7.78), but strawberry achieved the highest purchase intention (90.18%). Raspberry also generated a slightly higher positive-buying percentage than blackcurrant B despite a lower overall acceptance score. Purchase intention therefore reflected sensory acceptance but also contained additional decision components that were not measured directly, such as familiarity with the fruit, perceived product novelty, expected price, perceived health value, and confidence that the product would be chosen in a real purchasing situation.

Strawberry may have benefited from high familiarity, a recognizable aroma, and an expected flavor profile. Blackcurrant B, by contrast, was the best-liked formulation and combined high taste, color, consistency, and overall-acceptance ratings with a reduced-energy, erythritol-sweetened composition. Its positive purchase intention of nearly 80% indicates strong commercial potential, although the gap between liking and purchase intention suggests that product positioning and communication may still influence conversion from acceptance to buying.

For haskap berry, the large undecided and negative-response fractions suggest a need to optimize the sweetness–acidity balance, aroma, and visual saturation. Apple also requires substantial optimization, particularly in color, structure, and overall sensory distinctiveness. Plum had moderate overall acceptance and purchase intention, indicating a potentially viable niche formulation but weaker mass-market potential than strawberry, raspberry, or the blackcurrant variants.

### 2.9. Color Analysis and Consumer Color Acceptance of Probiotic Fruit Sorbets

The instrumental color analysis included CIELAB lightness (L*), redness/greenness (a*), yellowness/blueness (b*), chroma (C*), hue angle (*h*°) and within-matrix *ΔE*. Results are expressed as mean ± standard deviation of three analytical determinations. Tukey groups were calculated after one-way ANOVA, with the limitation that all analytical replicates originated from one production batch. High L* values describe lighter products. Positive a* values indicate redness, whereas negative values indicate a green component. Positive b* values indicate yellowness and negative values indicate a blue component. C* describes saturation, with higher values corresponding to more vivid color. Hue angle integrates the direction of a* and b* and should be interpreted jointly with those coordinates.

The significant Friedman result (Table 15) demonstrated that consumers discriminated among formulations on color. Color therefore contributed meaningfully to product perception. The analytical differences in sorbets’ lightness (L*), red-green coordinate (a*), yellow-blue coordinate (b*), color saturation (C*), hue angle (*h*°) were highly significant (*p* < 0.001). For within-matrix color variation (*ΔE*), the highest mean was observed for Haskap berry (2.72), while the lowest was recorded for Apple (0.50). The ANOVA result was *p* = 0.130. Consumer color ratings are presented in Table 16; both blackcurrant variants, raspberry and strawberry achieved the highest mean rating above 7 points, while the strawberry also received the highest median (9 points).

Consumer color ratings differed among sorbets (Friedman χ^2^(6) = 170.86, *p* < 0.001, Kendall’s W = 0.243) (Table 8). The highest mean color score was obtained by blackcurrant b (8.05), and the lowest by apple (5.93).

Instrumental color parameters showed different degrees of association with mean consumer color ratings. The strongest relationship was observed for redness, a*, which was strongly and positively correlated with consumer color acceptance according to Pearson’s analysis (r = 0.938, *p* = 0.002) (Table 17). Thus, sorbets with a stronger red component generally received higher color ratings. The Spearman coefficient showed a similar positive tendency (ρ = 0.750), although it narrowly failed to reach statistical significance (*p* = 0.052). Hue angle, *h*°, was strongly and negatively correlated with consumer color ratings in the Pearson analysis (r = −0.851, *p* = 0.015). This indicates that formulations shifted towards yellow tones received lower color ratings, whereas redder and more purple-toned sorbets were generally preferred. However, the corresponding Spearman correlation was weaker and non-significant. No statistically significant correlations were found for lightness, L*; yellowness, b*; or chroma, C*. Lightness and b* showed moderate negative tendencies, while chroma showed a weak-to-moderate positive association. Overall, the results suggest that the direction of color—particularly redness and hue—was more relevant to consumer preference than brightness or color saturation.

## 3. Discussion

The present study shows that fruit sorbets can serve as non-dairy carriers of *Lactiplantibacillus plantarum* 299v while providing inulin and fruit-derived bioactive compounds. The high post-freezing viability across seven chemically distinct fruit matrices supports the technological feasibility of this approach, whereas differences in texture, color, antioxidant properties, sensory acceptance, and purchase intention confirm that product quality remains strongly matrix- and sweetener-dependent. Few previous studies have combined a clinically characterized strain, a constant inulin dose, several fruit matrices, instrumental quality, antioxidant composition, consumer response, and purchase intention within one design [7,10,12,13,14]. The recipe developed in this study is so simple that it can be easily used to make sorbets with functional and potentially synbiotic properties at home. It is also a ready-made solution for artisan producers.

The use of *L. plantarum* 299v is important because probiotic effects are strain-specific. This strain is comparatively well characterized, particularly in relation to gastrointestinal function and host interaction [16,17,18,19]. Clinical studies have reported improvements in selected gastrointestinal symptoms in patients with irritable bowel [20] and some nutritional and gastrointestinal outcomes in cancer patients receiving enteral nutrition, although effects were not uniform [18]. The present work appropriately addresses the essential prerequisite for such effects: delivery of viable cells. The capsules used in the study are not only well-documented in earlier scientific research in terms of their effects on the human body, but also easily available. The use of pre-packaged probiotic capsules in pre-made ice cream mix before freezing is very simple and worthwhile.

Counts after freezing remained high in both inoculum variants, indicating good immediate tolerance of the process. This is notable because the sorbets were fat-free, acidic systems in which cell protection depended mainly on fruit solids, sugars, inulin, and the unfrozen aqueous phase. In avocado-based non-dairy ice cream, 299v also remained above the commonly applied probiotic threshold, but the lipid- and polysaccharide-rich matrix may have provided stronger cryoprotection [7]. The present findings therefore extend evidence of 299v survival to leaner and compositionally diverse fruit systems.

Two-capsule formulations produced higher final counts, although some showed a larger proportional reduction. Thus, a higher inoculum increased the post-freezing safety margin but was not necessarily more efficient. Acidity alone did not explain survival, suggesting that soluble solids, sugars, pectin, organic acids, polyphenols, oxygen exposure, and water mobility acted jointly. Similar matrix- and strain-dependent effects have been observed in riceberry-based frozen desserts containing inulin [10]. Long-term frozen storage studies are still needed because immediate survival does not establish shelf-life stability.

All formulations contained 3 g inulin/100 g. Inulin is fermented by intestinal microorganisms and is most consistently associated with a bifidogenic response, although the effect varies with dose, polymerization, background diet, microbiota, and host factors [3,4,27]. Nevertheless, the term synbiotic should be used cautiously: the present study did not demonstrate selective utilization of this inulin by 299v in the product or host. Because such interactions are strain- and substrate-dependent [5,6], the products are more precisely described as synbiotic-designed or potentially synbiotic sorbets.

Inulin may also contribute technologically by binding water, increasing serum-phase viscosity, reducing water mobility, and modifying ice formation. Improved viscosity, melting resistance, overrun, probiotic survival, and sensory quality have been reported in inulin-containing frozen yogurt and ice cream [11,29], while fructooligosaccharides similarly supported structure and probiotic viability in synbiotic dairy ice cream [12]. However, because all sorbets contained the same inulin concentration and no fiber-free control was included, its individual effect could not be isolated. Differences among products therefore mainly reflect interactions involving fruit solids, pectin, sugars, acids, water, and sweetener type. This agrees with earlier work showing that inulin modifies sorbet properties without overriding the dominant influence of fruit identity [2].

Fruit selection was the main determinant of vitamin C, anthocyanins, and antiradical activity. Haskap berry showed the highest functional density, and blackcurrant also provided high pigment-related and antioxidant values; raspberry and strawberry formed a second highly active group. This ranking is consistent with the phytochemical profiles of these berries [30,33,34,37,38,64,65]. Haskap is particularly rich in anthocyanins, iridoids, phenolic acids, flavonols, and vitamin C [35,36,37], whereas blackcurrant contains abundant delphinidin- and cyanidin-based anthocyanins [38,39].

Antiradical assays describe chemical reducing capacity rather than clinical efficacy and do not account for digestion, bioavailability, metabolism, or dose. The lower values of apple and plum should likewise not be interpreted as absence of nutritional value, because these fruits supply chlorogenic acids, flavonoids, procyanidins, triterpenoids, pectin, and other phenolics associated with potential health effects [40,41,42,43]. Future studies should combine complementary antioxidant assays, targeted phenolic profiling, in vitro digestion, and storage monitoring of vitamin C and anthocyanins.

The comparison of the two blackcurrant formulations provides the clearest evidence of sweetener effects. Replacing sucrose with erythritol reduced energy value and eliminated detectable sucrose while producing the highest hardness and lowest overrun. Despite these changes, blackcurrant B achieved very high acceptance and significantly greater purchase intention than the sucrose-sweetened counterpart. The result indicates that the intense acidity, aroma, and dark color of blackcurrant can accommodate erythritol-related changes in freezing behavior, solids balance, sweetness profile, and cooling sensation. It also shows that a dense texture is not inherently undesirable when it is congruent with fruit character. The sorbets’ hardness ranged between 5626 for apple and 7287 N for blackcurrant B, and those results were lower than berry craft sorbet with inulin studied by Leahu et al. [66]. The hardness of their sorbets varied from 7651–9568 N depending on inulin amount addition.

Hardness, overrun, and melting varied widely because frozen-dessert structure depends on the interaction of ice crystals, unfrozen serum, dissolved solids, fruit particles, pectin, and air. Apple combined the lowest hardness with the greatest melting resistance, whereas haskap was relatively hard but melted rapidly, confirming that penetration hardness and structural stability during warming describe different phenomena. Although inulin-enriched frozen desserts often show both increased viscosity and slower melting, this relationship is not universal across matrices [11,29]. Objective ice-crystal imaging, rheology, freezing curves, and glass-transition measurements would help explain these differences.

Instrumental color primarily reflected fruit identity, but it also contributed to sensory differentiation. Vivid red raspberry and strawberry and dark-purple blackcurrant products were visually well accepted, whereas the pale apple sorbet received a low color score. Similar anthocyanin contents but different color coordinates in the two blackcurrant variants indicate that pigment concentration alone does not determine appearance; ice fraction, air content, particle size, pH, copigmentation, dissolved solids, and light scattering also contribute. Correlations between color coordinates and acceptance should remain exploratory because they were based on only seven formulation means.

All sensory attributes differed among sorbets, and taste was most strongly associated with overall acceptance, followed by structure and consistency. Thus, high antioxidant potential or a functional claim cannot compensate for inadequate flavor and texture. Similar multidimensional effects of acidity, sweetness, creaminess, iciness, and flavor intensity have been reported in probiotic frozen desserts [12]. The non-fermented addition of 299v may be advantageous because it avoids further acidification after inoculation.

Blackcurrant B, raspberry, strawberry, and blackcurrant A were the most accepted products, with strawberry, raspberry, and blackcurrant B also showing the strongest commercial potential. Haskap illustrates the trade-off between functionality and market response: it had the highest bioactive density but lower liking and purchase intention, probably because of its acidity, characteristic aroma, and very dark appearance. Apple was least accepted, suggesting that familiarity alone was insufficient when color, aroma intensity, and structure were less attractive. After Holm correction for multiple comparisons, strawberry, raspberry, and blackcurrant B did not differ significantly from one another in purchase intention, supporting their interpretation as the highest-performing group. However, overlap was observed between adjacent groups, as blackcurrant A did not differ significantly from raspberry or plum.

Earlier studies demonstrated probiotic survival in individual pumpkin, jussara, mango, or other frozen matrices, but generally assessed fewer endpoints or only one raw material. Pumpkin sorbets containing inulin supported *L. rhamnosus* during frozen storage [14], whereas jussara sorbets retained bioactive compounds and probiotics but showed substantial losses during simulated gastrointestinal exposure [13]. By applying one strain and one inulin concentration across seven fruits, the present study links viability with physicochemical, antioxidant, sensory, and purchasing outcomes and distinguishes three useful strategies: high-bioactive matrices such as haskap and blackcurrant, familiar consumer-oriented matrices such as strawberry and raspberry, and the reduced-energy erythritol-sweetened blackcurrant B formulation.

Overall, the results support fruit sorbets as non-dairy carriers of viable *L. plantarum* 299v, inulin, and fruit bioactives. The working hypothesis was supported for immediate post-freezing survival, but confirmation of probiotic and bioactive stability during storage and evidence of a specific synbiotic interaction are still required. Successful formulation depends on balancing microbial viability, prebiotic potential, fruit composition, frozen structure, sensory quality, and consumer value. Among the tested products, blackcurrant B offered the most balanced profile, whereas haskap maximized bioactive content and strawberry and raspberry provided the strongest consumer-oriented performance.

## 4. Materials and Methods

### 4.1. Raw Materials for Synbiotic Functional Sorbets Preparation

The research materials were sorbets made of selected fruits: raspberry, strawberry, blackcurrant, haskap berry, apple, and plum, with the addition of water, sucrose (or erythritol) and inulin. Two blackcurrant sorbets were prepared; in one of the blackcurrant sorbets, sucrose was replaced with erythritol. The purpose of this substitution was to assess the effect of the sucrose substitute on the sorbet’s physicochemical and functional properties, the survival of probiotic bacteria, and to assess consumer acceptance. Blackcurrant was chosen for this experiment due to its high functional properties and gaining popularity in the market. This fruit was once very popular among consumers, but over time its popularity has declined. Consumer awareness has increased in recent years, and this fruit is becoming popular again due to its high health benefits. The basic ingredients of the sorbets were fresh fruits, obtained at edible ripeness; the choice was guided by popularity and availability of fruits and by their nutritional value. Inulin and erythritol were purchased in a local health food store; sucrose and low-mineralized mineral water were purchased in a local supermarket. The fruits were purchased directly from farmers growing fruit in the Kaszuby region, in the Pomeranian Voivodeship. Sorbet matrices were calculated according to gelato and sorbet composition; the dry substance content was crucial due to freezing process parameters and scraped-surface freezer characteristics. The recipes were primarily tested in small amounts. Too small a water content resulted in too-fast freezing of the matrix on the cylinder wall in the freezer. The detailed composition of the sorbets is given in Table 18.

The main goal was to develop sorbets composed of the fewest possible number of ingredients in the matrix. Therefore, lemon juice, commonly used in sorbet production, was removed from the recipe and only fruit, water, sucrose (or erythritol), and inulin were used. The addition of water to fruit during sorbet production is common, dictated not only by economic considerations but primarily by achieving the appropriate structure and consistency of the finished product. Seven types of sorbets were produced with the addition of one Sanprobi IBS capsule containing 1 × 10^10^ CFU *Lactiplantibacillus plantarum* 299v (SANPROBI sp. z o.o. sp. k., Szczecin, Poland). Another seven with the addition of two capsules (2 × 10^10^ CFU) were produced solely for investigation of the survival of probiotic bacteria and to assess the effect of the number of capsules on the content of these bacteria in the final product.

### 4.2. Preparation of the Sorbets

All sorbets were made with various fruits, water, sugar (or erythritol), and inulin. All ingredients including the probiotic capsule (two capsules in the two-capsule variant) (Table 18) were mixed in a Thermomix TM6 for 5 min at room temperature (first 40 s at maximum speed, then on low speed-level 3, about 500 rpm). Each sorbet mixture was mixed separately, then frozen in a Frigomat Twin 4 scraped-surface freezer (the temperature of the freezer was about −20 °C, time of freezing was about 4–5 min, and the temperature of the product upon leaving the freezer was about −4 to −5 °C). Then the sorbets were first hardened at −35 °C for 30 min and then stored at −25 °C in the Gdynia Maritime University laboratory for evaluation. The tests were performed after 24 h of storage.

### 4.3. Physico-Chemical Parameters

Extract Preparation for spectrophotometric methods. 5 g of the product was poured into 30 cm^3^ of 80% ethanol (1 h at room temperature, protected from light). The extract was centrifuged (20 min at 2000 rpm). The centrifuged extract was transferred to a 50 cm^3^ volumetric flask and adjusted to the desired volume with 80% alcohol. The resulting extract was used to determine the content of polyphenolic compounds, the ability to reduce free radicals, FRAP assay, and the content of anthocyanin pigments.

Total polyphenols content TP (mg GAE/g) was determined using Folin–Ciocalteu reagent and a standard curve of Gallic acid (AR) and results were expressed as mg Gallic acid equivalent (GAE)/1 g sample [67]. A calibration curve for the standard solution, gallic acid, was prepared at the appropriate concentrations. The prepared solutions for measurement (0.1 mL of each calibration solution, 6 mL of distilled water, and 0.5 mL of Folin–Ciocalteu reagent) were mixed and left for 3 min. 1.5 mL of saturated sodium carbonate solution was added, made up to the mark with distilled water, and mixed. Incubated for 30 min at 40 °C. Absorbance was measured at 765 nm against a blank (0 mg/mL gallic acid solution). Tested sorbet samples: procedure as above; extract solutions were taken instead of the calibration solution.

Antiradical activity AA (%) was estimated as free radical scavenging activity using 1,1-diphenyl-2-picryl-hydrazil (DPPH) [67,68]. 20 mm^3^ of the extract was added to 1.5 cm^3^ of the DPPH reagent and incubated for 30 min at 30 °C. The absorbance of the samples was measured at a wavelength of 517 nm.

FRAP assay, as described by Benzie and Strain [69]. The FRAP-2,4,6-tri(2-pyridyl)-s-triazine (TPTZ) reagent was prepared from the sodium acetate buffer (300 mM, pH 3.6), 10 mM 2,4,6-tri(2-pyridyl)-s-triazine (TPTZ) solution (40 mM HCl as solvent), and 20 mM FeCl3·6H2O in a volume ratio of 10:1:1. The FRAP reagent was freshly prepared on the day of the measurements. An aliquot of 75 μL of the extract was mixed with 225 μL of water and 2250 μL of FRAP reagent (pre-incubated for 5 min at 25 °C). The absorbance of the reagent mixture was measured at 593 nm after 30 min incubation at 25 °C.

The content of anthocyanin pigments was determined spectrophotometrically using the differential method according to Fuleki and Francis [70]. Absorbance was measured at 530 and 657 nm and the TMA content was expressed in mg of cyanidin-3-glucoside(C3G) equivalent per 100 g of fresh weight.

Vitamin C content analysis was performed using a modification of the Polish Standard PN-EN 14130:2003 Foodstuffs. Vitamin C Determination by HPLC According to [71].

Melting resistance (%) was measured by determining the amount of melted sorbets from a given sample volume; the method is described by [2].

The overrun was measured by determining the amount of air in a given volume of the sample; the method is described by [2].

Active acidity (pH) was determined according to the IUPAC Recommendations 2002 [72].

Titratable acidity (°SH) was determined according to the AOAC method [73].

Sugar content (fructose, glucose, sucrose) was analyzed using high-performance liquid chromatography (HPLC) in an isocratic system with light scattering detection (ELSD)–C18 column, flow rate 0.7 mL/min, sample injection 10 μL, mobile phase: 0.01% acetic acid: methanol (95:5, *v*/*v*).

Extract (°Brix) was determined by the refractometric method [74].

Fibre (g) was determined according to [75].

Dry matter content (%) analysis was performed using the oven-drying method. Samples weighing approximately 10 g were mixed with sand and dried at 70 °C for 2 h (after an hour, the samples were mixed). Subsequently, they were dried at 105 °C for approximately 3 h. After being placed in a desiccator, they were weighed and dried for another hour at 103 °C.

Ash content analysis was performed in accordance with the Polish Standard PN-EN 1135:1999 Foodstuffs [76].

Hardness was evaluated using a Brookfield CT3 TA (AMETEK, Berwyn, PA, USA) as a standard single test and expressed as the maximum load recorded during the test [N]. Texture analysis was conducted with TA10 (cylinder 12.7 mm in diameter, height 35 mm) at 0.05 N and a test speed of 1 mm/s. The cylindrical probe touched the surface of the sorbet and then penetrated the samples for five seconds. The measurements were conducted on sorbets at −15 °C in 1 L containers with an 80 mm depth.

Heat of combustion analysis was performed using a KL-12Mn calorimeter from PRECYZJA-BIT (Bydgoszcz, Poland) (so-called bomb calorimeter). The apparatus consists of a container into which the test sample is placed, filled with oxygen, and then ignited. The heat of combustion at constant volume is determined from the temperature increase.

All analyses were conducted three times.

### 4.4. Lactiplantibacillus plantarum 299v Viability Before and After Freezing

Twenty-gram samples of the products were collected under sterile conditions and then homogenized with 180 mL of buffered peptone water (Merck, Darmstadt, Germany). From this dilution, a serial dilution of up to 10^−8^ was carried out. 1 mL of the dilution was inoculated onto MRS Agar (Oxoid, Ltd., Hampshire, UK) using the pour-plate technique. The cultures were incubated at 30 °C for 72 h under microaerophilic conditions. According to the user’s manual, light-straw colonies were classified as presumptive LAB/*Lactiplantibacillus* and described in the manuscript as (most probably) *Lactiplantibacillus plantarum* 299v. Their counts were then determined according to the Polish Standard PN-EN ISO 7218:2024-12 [77].

### 4.5. Color Analysis

Color measurements of sorbets were carried out using the CIELab system. The obtained results were expressed in terms of CIE *L*, *a*, and *b* values. *L* indicates brightness, *a* represents red to green coordinates, and *b* represents the blue to yellow coordinates of a product [78]. The color of the sorbets was determined using a Konica Minolta CM–5 spectrophotometer (Konica Minolta Sensing, Osaka, Japan). Measurements were made at 5 different locations on the surface of the sorbets. Average color parameters were determined, and the Euclidean distance between two points in the three–dimensional space defined by *L*, *a*, and *b* was used to calculate the color difference between samples. The differences in color (*ΔE*) between variations relative to the mean color within each formulation were calculated from the formula [78]:

(1) $$\Delta E=\sqrt{\Delta L^{2}+\Delta a^{2}+\Delta b^{2}}$$

where

*ΔL* = brightness difference,

*Δa* = redness difference, and

*Δb* = yellowness difference.

Based on the *a* and *b* values, chroma, *C*, was calculated according to the formula [79]:

(2) $$C=\sqrt{a^{2}+b^{2}}$$

The hue angle, *h*°, was calculated according to the equation [79]:

(3) h°=arctanb/a

The hue angle result was converted to degrees and expressed in a range from 0 to 360°. The overall color difference between the selected sorbet variants was calculated based on the mean *L*, *a*, and *b* values according to the CIE formula:

(4) $$\Delta E=\sqrt{\Delta L^{2}+\Delta a^{2}+\Delta b^{2}}$$

where *ΔL*, *Δa*, and *Δb* represented the differences between the mean values of the corresponding color coordinates of the compared sorbets. The *ΔEab* values were used to assess the magnitude of the visual difference between the samples.

When comparing two sorbet variants, normality of distribution and homogeneity of variance were assessed before selecting the statistical test. Due to the small sample size and the possibility of unequal variances, the *L*, *a*, and *b* values were compared using Welch’s two-tailed *t*-test for independent samples. To simultaneously assess differences in the overall color profile, including the *L*, *a*, and *b* coordinates, the Hotelling T^2^ test was used. When comparing three sorbet variants, a one-way ANOVA was used separately for the *L*, *a*, and *b* parameters. Once a significant effect of sorbet type was detected, a Tukey post hoc test was performed to identify pairs of variants that statistically differed. The combined effect of sorbet type on the set of *L*, *a*, and *b* coordinates was assessed using a multivariate analysis of variance (MANOVA) with Wilks’ lambda statistic. Additional pairwise comparisons of the overall color profiles were performed using the Hotelling T^2^ test. The level of statistical significance was set at *p* < 0.05. The results of five measurements were averaged and the test was repeated three times.

### 4.6. Evaluation of Consumer Acceptance and Purchase Intention

The coded samples were served to panellists under normal daylight. Due to the recommendation to evaluate a maximum of five products at a time, the study was conducted at different times of day, as recommended in the sensory analysis. Between 9 a.m. and 10 a.m., each panelist was presented with four sorbets for evaluation. After a break, between 12 p.m. and 1 p.m., three sorbets were served. Between each taste, panelists rinsed their mouths with water, and a baguette was also provided to neutralize the taste. Staff and students of Gdynia Maritime University were invited to participate in the evaluation. The study group consisted of 57 men and 60 women, aged 21 to 55.

Each panelist received samples at approximately −12 °C for evaluation (that temperature is recommended when serving ice cream or sorbet). The consumer acceptance and purchase intention investigation took place in a laboratory in Gdynia Maritime University, Poland. Respondents rated all sorbet variants. The acceptance and purchase intention were tested on 117 consumer panellists. Samples of about 30 g of each product were served with code numbers; samples were served at approximately −12 °C for evaluation (this temperature is recommended when serving sorbet or ice cream). Each participant evaluated all product variants, so the data obtained were dependent. In this research, a preference test card (Preference Test) was used. General appearance, color, taste, odor, consistency, structure and overall acceptability were assessed using a 9-point hedonic scale, with 1 indicating the lowest and 9 indicating the highest level of acceptability (9 for Like Extremely, 8 for Like Very Much, 7 for Like Moderately, 6 for Like Slightly, 5 for Neither Like nor Dislike, 4 for Dislike Slightly, 3 for Dislike Moderately, 2 for Dislike Very Much and 1 for Dislike Extremely). The purchase intention was evaluated using a 5-point scale (by answering the question: “Would you buy this sorbet?” with one of the proposed answers: No; Probably no; I don’t know; Probably yes; Yes. Human participants were recruited during the study on 1 June 2026, and the study was completed on 5 June 2026. All participants provided written informed consent each time sensory evaluation was performed. The study did not include minors. The cards used for sensory evaluation each time included a statement that the evaluator consented to participate in the study. After completing the consumer evaluation, the results were exported to a Microsoft Excel spreadsheet and statistically analysed. The arithmetic mean, standard deviation, median, interquartile range, and minimum and maximum values were calculated. Sensory ratings were analyzed using repeated-measures ANOVA with Tukey-adjusted comparisons and Friedman tests with Kendall’s W. Purchase intention was analyzed using the Friedman test followed by paired Wilcoxon signed-rank tests with Holm correction for multiple comparisons. Additionally, the percentage of positive ratings was determined, using a score of ≥7 points as the threshold for acceptance, and a score of ≥8 points as the threshold for high acceptance. The relationships between sensory attribute ratings and overall acceptance were analysed using Spearman’s rank correlation coefficient. Results were considered statistically significant at *p* < 0.05 [80,81].

### 4.7. Statistical Analysis

Laboratory data were reported as mean ± SD of three analytical replicates from one production batch. Physicochemical, nutritional, antioxidant, textural, technological, and color variables were analyzed by one-way ANOVA with Tukey’s HSD test; values below the detection limit were treated as missing. *Lactiplantibacillus plantarum* 299v counts were analyzed using linear mixed-effects ANOVA with sorbet formulation, capsule number, freezing, and their interactions as fixed effects and replicate as a random intercept. Type III F-tests used Satterthwaite-adjusted degrees of freedom, while planned contrasts used Kenward–Roger adjustment and Holm correction. Log reduction was analyzed analogously; survival was not tested separately. Consumer acceptance and purchase-intention data were analyzed as described in Section 4.6. Additional Pearson and Spearman correlations were treated as explanatory. Statistical significance was set at *p* < 0.05. Analyses were performed in R 4.2.2 and Python 3.13.5; figures were generated with Matplotlib 3.10.8 [82,83,84,85,86].

## 5. Conclusions

The present study demonstrated that high-fruit, non-dairy sorbets can be developed as multifunctional potential carriers of the clinically characterized probiotic strain *Lactiplantibacillus plantarum* 299v, while simultaneously providing inulin and fruit-derived bioactive compounds. The comparative design showed that probiotic viability, physicochemical quality, health-promoting composition, consumer acceptance, and purchase intention can be evaluated within one coherent product-development framework. The hypotheses were verified positively. On the basis of microbiological analysis it can be stated that *Lactiplantibacillus plantarum* 299v most probably remained at high viable counts after freezing in all tested sorbet matrices. The results support technological feasibility, although long-term frozen-storage stability still requires confirmation. The two-capsule variants produced higher final viable counts, but did not consistently show a lower proportional loss. A higher starting dose increased the final safety margin rather than guaranteeing greater relative resistance. Fruit type and the amount in the matrix significantly differentiated the physicochemical, color, antioxidant, and sensory characteristics of the products. The results confirm that matrix composition is a major determinant of sorbet quality. Overall acceptance was most strongly associated with taste, structure, and consistency, with additional contributions from appearance, color, and odor. Purchase intention therefore depended on integrated sensory quality. Erythritol enabled the development of a lower-sucrose blackcurrant sorbet and preserved its principal functional features, but it modified freezing behavior and sensory properties. Further optimization is required before equivalence with the sucrose-sweetened formulation can be claimed.

The study fills an important gap because previous research on probiotic or synbiotic sorbets has generally been restricted to one raw material, one limited technological endpoint, or probiotic storage alone. The present work compared several high-fruit matrices under a common formulation strategy and integrated strain-specific survival, inulin content, bioactive compounds, physicochemical parameters, sensory acceptance, and purchase intention. This broad comparison provides a more realistic basis for selecting formulations for further development.

An important practical advantage of the developed products is the simplicity of their formulation. The recipes are based on readily available fruit, water, a sweetening component, inulin, and a commercially available preparation containing *Lactiplantibacillus plantarum* 299v. Consequently, the formulation concept can be transferred relatively easily to small-scale artisanal production and even to domestic preparation.

Future experiments should include several independently manufactured batches to quantify process variability. Viability should be monitored for at least 3–6 months under controlled and fluctuating temperatures and provide strain-specific confirmation, e.g., molecular identification. Developing technologies for industrial production of synbiotic, functional sorbets could also be a good direction for future research.

## Figures and Tables

**Table 1:** (**a**) Physicochemical parameters of sorbets. (**b**) Physicochemical parameters of sorbets. (**c**) Physicochemical parameters of sorbets. (**d**) Instrumental color parameters of sorbet.

(**a**)							
Matrix	Active acidity (pH)	Potential acidity (°SH)	Antiradical activity AA DPPH (%)	Antioxidant Activity FRAP (µmol TE/g)	Total polyphenols TP (mg GAE/g)	Total anthocyanins (mg/100 g)	Vitamin C (mg/100 g)
Raspberry	2.89 ± 0.012 c	5.57 ± 0.039 b	90.24 ± 1.90 ad	3.55 ± 0.11 d	7.04 ± 0.141 b	166.0 ± 8.9 c	16.07 ± 0.01 e
Strawberry	3.58 ± 0.032 a	2.76 ± 0.034 d	86.45 ± 0.29 d	2.57 ± 0.72 d	5.98 ± 0.053 d	144.0 ± 7.5 ce	42.82 ± 0.01 d
Blackcurrant A	2.86 ± 0.017 c	5.52 ± 0.039 b	92.47 ± 0.12 a	28.39 ± 13.16 bc	5.45 ± 0.058 e	1294.3 ± 9.3 b	65.33 ± 0.00 b
Blackcurrant B	2.74 ± 0.015 d	5.54 ± 0.056 b	91.66 ± 0.76 a	33.09 ± 12.74 c	5.44 ± 0.069 e	1294.3 ± 9.3 b	51.72 ± 0.01 c
Haskap berry	3.20 ± 0.012 b	9.43 ± 0.059 a	93.35 ± 0.68 a	26.17 ± 2.39 abc	6.55 ± 0.129 c	1808.3 ± 23.0 a	94.78 ± 0.51 a
Apple	3.18 ± 0.036 b	4.95 ± 0.229 c	57.56 ± 2.82 c	12.13 ± 2.44 abd	3.95 ± 0.049 f	125.3 ± 8.5 e	8.58 ± 0.229 f
Plum	2.90 ± 0.032 c	5.46 ± 0.026 b	65.54 ± 3.38 b	6.71 ± 0.3.40 ad	7.59 ± 0.044 a	86.0 ± 8.00 d	3.52 ± 0.345 g
(**b**)							
Matrix	Hardness (N)	Melting resistance (%)	Overrun (%)	Total dry solids (%)		Fiber (g)	Ash (%)
Raspberry	6509.3 ± 33.1 b	21.60 ± 0.36 c	36.03 ± 0.40 a	29.24 ± 0.17 d		0.82 ± 0.073 bd	0.22 ± 0.006 c
Strawberry	6346.0 ± 21.8 c	24.43 ± 0.47 f	35.57 ± 0.38 af	30.52 ± 0.12 c		1.12 ± 0.114 a	0.37 ± 0.010 b
Blackcurrant A	6280.0 ± 22.6 c	18.73 ± 0.23 d	33.97 ± 0.21 b	30.20 ± 0.09 c		0.54 ± 0.046 c	0.24 ± 0.005 c
Blackcurrant B	7287.3 ± 37.4 a	25.87 ± 0.49 b	19.20 ± 0.40 e	25.52 ± 0.72 e		0.55 ± 0.022 c	0.25 ± 0.023 c
Haskap berry	6560.7 ± 119.1 b	14.67 ± 0.31 e	24.90 ± 0.53 d	34.53 ± 0.14 a		0.70 ± 0.067 cd	0.52 ± 0.019 a
Apple	5628.3 ± 27.3 d	30.47 ± 0.47 a	34.87 ± 0.31 bf	32.63 ± 0.22 b		0.63 ± 0.031 c	0.14 ± 0.005 d
Plum	6201.7 ± 36.5 c	25.50 ± 0.26 bf	28.70 ± 0.36 c	32.55 ± 0.14 b		0.94 ± 0.037 b	0.22 ± 0.007 c
(**c**)							
Matrix	Extract (Brix)	Fructose (g/100 g)	Glucose (g/100 g)	Sucrose (g/100 g)		Heat of combustion (kJ/g dw)	
Raspberry	21.80 ± 0.10 c	2.48 ± 0.010 d	2.31 ± 0.000 e	19.18 ± 0.01 c		17.10 ± 0.12 ad	
Strawberry	30.30 ± 0.20 b	2.45 ± 0.012 e	9.83 ± 0.010 b	8.22 ± 0.012 d		16.08 ± 0.19 ce	
Blackcurrant A	18.77 ± 0.12 d	2.75 ± 0.000 c	2.75 ± 0.012 d	19.65 ± 0.01 b		15.95 ± 0.30 cg	
Blackcurrant B	17.87 ± 0.06 e	2.27 ± 0.000 f	1.97 ± 0.010 f	BDL		17.38 ± 0.17 a	
Haskap berry	35.80 ± 0.10 a	7.25 ± 0.000 a	15.58 ± 0.01 a	4.81 ± 0.012 f		15.97 ± 0.23 cf	
Apple	22.13 ± 0.21 c	4.17 ± 0.010 b	1.27 ± 0.012 g	8.03 ± 0.012 e		16.61 ± 0.12 bd	
Plum	21.83 ± 0.15 c	1.40 ± 0.010 g	6.07 ± 0.000 c	20.26 ± 0.02 a		16.16 ± 0.01 befg	
(**d**)							
Matrix	*L*	*a*	*b*	*ΔEab* relative to the variant mean		*C*	*h*°
Raspberry	38.57 ± 1.04 b	38.04 ± 0.90 a	19.48 ± 1.32 b	1.48 ± 0.61 a		42.75 ± 1.38 a	27.10 ± 1.09 d
Strawberry	39.96 ± 0.79 b	34.21 ± 1.06 b	24.70 ± 2.25 e	1.89 ± 1.21 a		42.21 ± 2.16 a	35.77 ± 1.67 c
Blackcurrant A	16.37 ± 0.09 c	29.85 ± 1.12 c	13.18 ± 1.91 c	1.71 ± 0.73 a		32.65 ± 1.80 b	23.74 ± 2.22 df
Blackcurrant B	13.47 ± 1.05 c	28.26 ± 1.20 c	11.07 ± 1.54 c	1.63 ± 0.97 a		30.36 ± 1.67 b	21.33 ± 1.91 ef
Haskap berry	13.92 ± 2.73 c	14.34 ± 1.89 d	5.07 ± 1.046 d	2.72 ± 1.00 a		15.21 ± 2.13 c	19.37 ± 1.27 e
Apple	48.90 ± 0.11 a	4.38 ± 0.200 f	31.16 ± 0.62 a	0.50 ± 0.23 a		31.46 ± 0.62 b	82.00 ± 0.37 a
Plum	47.50 ± 1.40 a	10.09 ± 0.36 e	27.46 ± 1.20 ae	1.52 ± 0.25 a		29.26 ± 1.04 b	69.80 ± 1.38 b

Table 1a–d: different letters within a column indicate significant differences at *p* ≤ 0.05 according to one-way ANOVA followed by Tukey’s HSD. The letters describe analytical separation within one production batch. BDL, below detection limit.

**Table 2:** Viability of *Lactiplantibacillus plantarum* 299v before and after freezing in fruit sorbets.

Sorbet Matrix	Capsules	Before Freezing (log CFU/g) Mean ± SD	After Freezing (log CFU/g) Mean ± SD	Log Reduction	Apparent Survival (%)
Raspberry	1	7.11 ± 0.03	7.05 ± 0.02	0.06	87.47
Raspberry	2	7.63 ± 0.02	7.49 ± 0.02	0.15	71.82
Strawberry	1	7.17 ± 0.02	7.17 ± 0.05	0.00	99.85
Strawberry	2	7.56 ± 0.12	7.46 ± 0.05	0.11	79.53
Blackcurrant A	1	7.13 ± 0.02	7.08 ± 0.04	0.05	90.26
Blackcurrant A	2	7.63 ± 0.06	7.49 ± 0.02	0.15	71.82
Blackcurrant B	1	7.01 ± 0.02	7.05 ± 0.02	−0.04	109.65
Blackcurrant B	2	7.59 ± 0.02	7.51 ± 0.00	0.08	82.41
Haskap berry	1	7.17 ± 0.02	7.15 ± 0.03	0.02	95.41
Haskap berry	2	7.51 ± 0.00	7.45 ± 0.02	0.06	87.50
Apple	1	7.19 ± 0.02	7.18 ± 0.03	0.01	98.05
Apple	2	7.70 ± 0.00	7.51 ± 0.00	0.19	63.97
Plum	1	7.07 ± 0.02	7.09 ± 0.02	−0.02	105.85
Plum	2	7.63 ± 0.11	7.49 ± 0.02	0.15	71.82

Mean values were calculated from three replicates (three different portions of sorbets were used for the analysis).

**Table 3:** (**a**) Exploratory statistical comparison of *Lactiplantibacillus plantarum* 299v viability according to type of the sorbet, probiotic dose and freezing. (**b**) Before- vs. after-capsule *Lactiplantibacillus plantarum* 299v viability comparison. (**c**) One- vs. two-capsule *Lactiplantibacillus plantarum* 299v viability comparison. (**d**) Log reduction and survival rate of *Lactiplantibacillus plantarum* 299v. (**e**) *Lactiplantibacillus plantarum* 299v survival rate in the one-capsule condition.

(**a**)						
Effect	F	df	*p*-value		Conclusion	
Sorbet type	3.3217	6.14	0.03008		Significant	
Capsule number	1880.9392	1.42	<0.0001		Significant	
Freezing	47.7125	1.42	<0.0001		Significant	
Sorbet × capsule number	7.8022	6.42	<0.0001		Significant	
Sorbet × freezing	1.5757	6.42	0.17813		Not significant	
Capsule number × freezing	34.1955	1.42	<0.0001		Significant	
Sorbet × capsule number × freezing	0.9170	6.42	0.49236		Not significant	
(**b**)						
estimate = before–after (freezing mean)						
Capsule	Estimate	SE	df	t	*p*-value	Conclusion
One	0.0105	0.014	42	0.749	0.4578	Not significant
Two	0.1267	0.014	42	9.019	<0.0001	Significant
(**c**)						
estimate = two–one (capsule(s) mean)						
Freezing	Estimate	SE	df	t	*p*-value	Conclusion
Before	0.489	0.014	42	34.802	<0.0001	Significant
After	0.373	0.014	42	26.532	<0.0001	Significant
(**d**)						
Effect	F	df	*p*-value		Conclusion	
Sorbet type	1.8474	6.14	0.16126		Not significant	
Capsule number	114.3150	1.14	<0.0001		Significant	
Sorbet × capsule number	3.0655	1.14	0.03944		Significant	
(**e**)						
estimate = one–two (capsule(s) mean)						
Estimate	SE	df	t	*p*-value	Conclusion	
−0.116	0.0109	14	−10.692	<0.0001	Significant	

**Table 4:** Matrix-level correlations between pH and log reduction.

Capsules	Pearson r	Pearson *p*	Spearman rho	Spearman *p*
1	0.051	0.913	0.036	0.939
2	<0.001	0.778	<0.001	0.812

**Table 5:** Results of omnibus repeated-measures ANOVA for consumer ratings.

Sensory Attribute	*F*-Value	Error df	*p*-Value
General appearance	26.48	696	<0.001
Color	35.42	696	<0.001
Taste	17.25	696	<0.001
Odor	15.53	696	<0.001
Consistency	22.00	696	<0.001
Structure	23.93	696	<0.001
Overall acceptance	25.65	696	<0.001

Repeated-measures ANOVA was applied because each participant evaluated all sorbet formulations. Sorbet type had a statistically significant effect on every evaluated sensory attribute.

**Table 6:** Consumer acceptance scores.

Sorbet	General Appearance	Color	Taste	Odor	Consistency	Structure	Overall Acceptance
Raspberry	7.76 ± 1.27 a	7.77 ± 1.51 a	7.60 ± 1.60 ad	7.41 ± 1.74 a	7.56 ± 1.63 a	7.50 ± 1.60 a	7.62 ± 1.61 a
Blackcurrant A	7.45 ± 1.57 a	7.73 ± 1.55 a	7.20 ± 1.92 bde	6.60 ± 1.69 b	7.47 ± 1.54 a	7.27 ± 1.61 a	7.25 ± 1.77 a
Haskap berry	6.50 ± 1.57 b	6.98 ± 1.61 b	6.37 ± 1.84 cf	6.44 ± 1.66 b	6.38 ± 1.71 bc	6.24 ± 1.61 bc	6.42 ± 1.69 bc
Strawberry	7.86 ± 1.54 a	7.97 ± 1.68 a	7.56 ± 1.90 ae	7.72 ± 1.75 a	7.40 ± 1.86 a	7.48 ± 1.81 a	7.57 ± 1.68 a
Plum	6.62 ± 2.00 b	6.29 ± 1.98 c	6.73 ± 2.13 bfg	6.63 ± 1.98 b	6.68 ± 1.91 b	6.63 ± 1.74 b	6.54 ± 1.80 b
Apple	6.05 ± 1.85 b	5.93 ± 1.92 c	6.25 ± 1.95 cg	6.21 ± 1.75 b	5.79 ± 1.83 c	5.71 ± 1.84 c	5.86 ± 1.77 c
Blackcurrant B	7.71 ± 1.20 a	8.05 ± 1.07 a	7.98 ± 1.03 a	7.44 ± 1.15 a	7.62 ± 1.15 a	7.45 ± 1.11 a	7.78 ± 1.01 a

Values are expressed as mean ± standard deviation (n = 117). Different letters within a column indicate significant differences between sorbet means at *p* ≤ 0.05 according to Tukey-adjusted comparisons following repeated-measures ANOVA.

**Table 7:** Pearson correlations between sensory attributes and overall acceptance.

Sensory Attribute	Pearson r	*p*-Value	Strength and Direction
General appearance	0.988	<0.001	Very strong positive
Structure	0.982	<0.001	Very strong positive
Consistency	0.980	<0.001	Very strong positive
Taste	0.975	<0.001	Very strong positive
Color	0.950	0.001	Very strong positive
Odor	0.892	0.007	Strong positive

**Table 8:** Friedman test results for sensory attributes.

Attribute	Chi-Square	df	*p*-Value	Kendall W
General appearance	132.830	6	<0.001	0.189
Color	170.857	6	<0.001	0.243
Taste	100.425	6	<0.001	0.143
Odor	95.622	6	<0.001	0.136
Consistency	116.417	6	<0.001	0.166
Structure	126.834	6	<0.001	0.181
Overall acceptance	138.067	6	<0.001	0.197

Differences among seven sorbet formulations were analyzed using the Friedman repeated-measures test. Kendall’s W was reported as a measure of effect size and consumer concordance. For all attributes, *p* < 0.001.

**Table 9:** Spearman correlations with overall acceptance.

Attribute	Spearman rho	*p*-Value
General appearance	0.738	<0.001
Color	0.752	<0.001
Taste	0.859	<0.001
Odor	0.749	<0.001
Consistency	0.817	<0.001
Structure	0.823	<0.001

**Table 10:** Differences in consumer scores between blackcurrant A and blackcurrant B.

Attribute	Blackcurrant A	Blackcurrant B	Difference, B − A
General appearance	7.45	7.71	+0.26
Color	7.73	8.05	+0.32
Taste	7.20	7.98	+0.78
Odor	6.60	7.44	+0.84
Consistency	7.47	7.62	+0.15
Structure	7.27	7.45	+0.18
Overall acceptance	7.25	7.78	+0.53

**Table 11:** Distribution of purchase-intention responses.

Sorbet	Valid n	No	Probably No	Undecided	Probably Yes	Yes	Mean	Median	Positive (%)
Raspberry	113	1	5	16	31	60	4.27	5.00	80.53
Blackcurrant A	114	6	7	16	43	42	3.95	4.00	74.56
Haskap berry	112	4	22	29	33	24	3.46	4.00	50.89
Strawberry	112	0	5	6	45	56	4.36	4.50	90.18
Plum	113	6	17	23	36	31	3.61	4.00	59.29
Apple	117	6	18	33	40	20	3.43	4.00	51.28
Blackcurrant B	114	0	3	20	47	44	4.16	4.00	79.82

**Table 12:** Friedman test results for purchase intention for 7 sorbet formulations; n = 98.

Outcome	Friedman χ^2^	df	*p*-Value	Kendall W
Purchase intention	102.47	6	<0.001	0.174

Purchase intention was evaluated on a five-category ordinal scale coded from 1 (“No”) to 5 (“Yes”). The Friedman test included only consumers with complete valid responses for all seven sorbet formulations. Kendall’s W was used as the effect-size measure.

**Table 13:** Pairwise comparisons without statistically significant differences.

Pair of the Sorbets	Raw *p*-Value	Holm-Adjusted *p*-Value
Raspberry vs. Strawberry	0.544	1.000
Raspberry vs. Blackcurrant B	0.333	0.998
Haskap berry vs. Plum	0.117	0.586
Haskap berry vs. Apple	1.000	1.000
Plum vs. Apple	0.128	0.586
Raspberry vs. Blackcurrant A	0.045	0.269
Plum vs. Blackcurrant A	0.026	0.184
Strawberry vs. Blackcurrant B	0.010	0.084

**Table 14:** Correlations between overall acceptance and purchase-intention indicators.

Variables	Method	Coefficient	*p*-Value
Overall acceptance vs. mean purchase-intention score	Pearson	0.948	0.001
Overall acceptance vs. mean purchase-intention score	Spearman	0.857	0.014
Overall acceptance vs. positive purchase intention (%)	Pearson	0.934	0.002
Overall acceptance vs. positive purchase intention (%)	Spearman	0.821	0.023

**Table 15:** ANOVA results for instrumental color parameters.

Parameter	F	*p*-Value
L*	439.528	0.000
a*	425.047	0.000
b*	120.641	0.000
C*	96.461	0.000
*h*°	836.295	0.000
Δ*E*	2.022	0.130

**Table 16:** Consumer color ratings.

Sorbet	n	Mean	SD	Median	Positive 7–9 (%)
Raspberry	117	7.77	1.51	8.00	84.62
Blackcurrant A	117	7.73	1.55	8.00	82.91
Haskap berry	117	6.98	1.61	7.00	61.54
Strawberry	117	7.97	1.68	9.00	87.18
Plum	117	6.29	1.98	6.00	49.57
Apple	117	5.93	1.92	6.00	37.61
Blackcurrant B	117	8.05	1.07	8.00	93.16

**Table 17:** Exploratory correlations between instrumental color and mean consumer color rating (n = 7).

Parameter	Pearson r	Pearson *p*	Spearman ρ	Spearman *p*
L*	−0.554	0.197	−0.643	0.119
a*	0.938	0.002	0.750	0.052
b*	−0.517	0.234	−0.500	0.253
C*	0.416	0.353	0.393	0.383
*h*°	−0.851	0.015	−0.500	0.253

**Table 18:** The composition of the sorbets (g/100 g).

Type of Fruit (Variety)	Fruits Amount	Water	Sucrose	Erythritol	Inulin	Sanprobi IBS *
Raspberry (*Glen*)	45.0	32.0	20.0	0.0	3.0	1 × 10^10^ CFU
Strawberry (*Rumba*)	55.0	32.0	10.0	0.0	3.0	1 × 10^10^ CFU
Blackcurrant A (*Gofert*)	45.0	32.0	20.0	0.0	3.0	1 × 10^10^ CFU
Blackcurrant B (*Gofert*)	45.0	32.0	0.0	20.0	3.0	1 × 10^10^ CFU
Haskap berry (*Duet*)	45.0	47.0	5.0	0.0	3.0	1 × 10^10^ CFU
Plum (*Węgierka Dąbrowicka*)	45.0	32.0	20.0	0.0	3.0	1 × 10^10^ CFU
Apple (*Szara Reneta/Grey Renet*)	55.0	32.0	10.0	0.0	3.0	1 × 10^10^ CFU

* *Lactiplantibacillus plantarum* 299v (1 capsule).

## Data Availability

The original contributions presented in this study are included in the article. Further inquiries can be directed to the author.

## Supplementary Materials

The following supporting information can be downloaded at: https://www.mdpi.com/article/10.3390/molecules31183258/s1, Table S1. Pairwise Wilcoxon signed-rank tests for purchase intention.

## Abbreviations

The following abbreviations are used in this manuscript:

log CFU	The base-10 logarithm (log10) of the number of colony-forming units (CFU)
°C	Celsius degrees
a*	Redness
b*	Yellowness
L*	Lightness
ΔE*	Difference in color
C*	Chroma
h°	Hue angle
°Brix	Brix degrees
°SH	Soxhlet–Henkel degrees
pH	Active acidity
n	Sample size
g	Gram
mg	Milligram
µmol	Micromol
DW	Dry weight
TP	Total polyphenols
DPPH	1,1-diphenyl-2-picryl-hydrazil
FRAP	Ferric reducing antioxidant power
GAE	Gallic acid equivalent
TE	Trolox equivalent
HPLC	High-performance liquid chromatography
kJ	Kilojoules
%	Percent
±	Plus-minus (standard deviation)
SD	Standard deviation
BDL	Below detection limit
df	Degrees of freedom
F	F-statistic
χ^2^	Chi-square statistical hypothesis test
Kendall W	Kendall’s coefficient of concordance
Pearson *p*	Pearson correlation coefficient
Pearson r	Pearson correlation coefficient
*p* < 0.05	Probability value less than 0.05 (statistical significance)
SE	Standard error
Spearman *p*	Spearman’s rank correlation coefficient
Spearman rho	Spearman’s rank correlation coefficient
t	T-statistic, Student’s *t*-test
