# Supplementary material for: Development of Functional Synbiotic-Designed Sorbets as Carriers of Lactiplantibacillus plantarum 299v and Fruit-Derived Bioactive Compounds: Probiotic Survival, Physicochemical Quality, Consumer Acceptance, and Purchase Intention
Source: Molecules. 2026 Sep 14;31(18):3258. doi: 10.3390/molecules31183258 (PMC13609199; doi:10.3390/molecules31183258)
Supplement: Supplementary file 1 [file molecules-31-03258-s001.zip › molecules-4508720-supplementary.pdf]

Table S1. Pairwise Wilcoxon signed-rank tests for purchase intention.

Sorbet 1	Sorbet 2	Paired valid n	Mean 1	Mean 2	Wilcoxon W	Raw p-value	Holm-adjusted p-value	Higher purchase intention	Significant
Raspberry	Blackcurrant A	110	4.28	3.96	1085.0	0.045	0.269	Raspberry	No
Raspberry	Haskap berry	108	4.31	3.46	444.0	<0.001	<0.001	Raspberry	Yes
Raspberry	Strawberry	108	4.29	4.35	835.5	0.544	1.000	None	No
Raspberry	Plum	110	4.26	3.65	807.0	<0.001	<0.001	Raspberry	Yes
Raspberry	Apple	113	4.27	3.42	535.0	<0.001	<0.001	Raspberry	Yes
Raspberry	Blackcurrant B	110	4.28	4.15	1247.0	0.333	0.998	None	No
Blackcurrant A	Haskap berry	109	3.94	3.42	777.0	<0.001	<0.001	Blackcurrant A	Yes
Blackcurrant A	Strawberry	109	3.94	4.39	546.0	<0.001	<0.001	Strawberry	Yes
Blackcurrant A	Plum	111	3.93	3.60	1134.5	0.026	0.184	Blackcurrant A	No
Blackcurrant A	Apple	114	3.95	3.39	648.5	<0.001	<0.001	Blackcurrant A	Yes
Blackcurrant A	Blackcurrant B	114	3.95	4.16	0.0	0.003	0.029	Blackcurrant B	Yes
Haskap berry	Strawberry	107	3.44	4.36	415.0	<0.001	<0.001	Strawberry	Yes
Haskap berry	Plum	108	3.41	3.61	1009.0	0.117	0.586	None	No
Haskap berry	Apple	112	3.46	3.43	1620.0	1.000	1.000	None	No
Haskap berry	Blackcurrant B	109	3.42	4.15	449.0	<0.001	<0.001	Blackcurrant B	Yes
Strawberry	Plum	108	4.34	3.64	692.0	<0.001	<0.001	Strawberry	Yes

Sorbet 1	Sorbet 2	Paired valid n	Mean 1	Mean 2	Wilcoxon W	Raw p-value	Holm-adjusted p-value	Higher purchase intention	Significant
Strawberry	Apple	112	4.36	3.40	379.5	<0.001	<0.001	Strawberry	Yes
Strawberry	Blackcurrant B	109	4.39	4.15	631.5	0.010	0.084	Strawberry	No
Plum	Apple	113	3.61	3.39	1343.5	0.128	0.586	None	No
Plum	Blackcurrant B	111	3.60	4.14	838.5	<0.001	<0.001	Blackcurrant B	Yes
Apple	Blackcurrant B	114	3.39	4.16	373.5	<0.001	<0.001	Blackcurrant B	Yes
